# Ultrasound-Promoted preparation and application of novel bifunctional core/shell Fe_3_O_4_@SiO_2_@PTS-APG as a robust catalyst in the expeditious synthesis of Hantzsch esters

**DOI:** 10.1038/s41598-023-33990-7

**Published:** 2023-05-17

**Authors:** Peyman Shakib, Mohammad G. Dekamin, Ehsan Valiey, Shahriar Karami, Mohammad Dohendou

**Affiliations:** Pharmaceutical and Heterocyclic Compounds Research Laboratory, Department of Chemistry, Iran University of Science and Technology, Tehran, 1684613114 Iran

**Keywords:** Drug screening, Medicinal chemistry, Catalysis, Energy, Environmental chemistry, Green chemistry, Inorganic chemistry, Materials chemistry, Medicinal chemistry, Organic chemistry, Surface chemistry, Drug discovery, Environmental sciences, Molecular medicine, Chemistry, Energy science and technology, Engineering

## Abstract

In this work, D-(–)-α-phenylglycine (APG)-functionalized magnetic nanocatalyst (Fe_3_O_4_@SiO_2_@PTS-APG) was designed and successfully prepared in order to implement the principles of green chemistry for the synthesis of polyhydroquinoline (PHQ) and 1,4-dihydropyridine (1,4-DHP) derivatives under ultrasonic irradiation in EtOH. After preparing of the nanocatalyst, its structure was confirmed by different spectroscopic methods or techniques including Fourier transform infrared (FTIR) spectroscopy, energy-dispersive X-ray spectroscopy (EDS), field emission scanning electron microscopy (FESEM), X-ray diffraction (XRD), vibrating sample magnetometer (VSM) and thermal gravimetric analysis (TGA). The performance of Fe_3_O_4_@SiO_2_@PTS-APG nanomaterial, as a heterogeneous catalyst for the Hantzsch condensation, was examined under ultrasonic irradiation and various conditions. The yield of products was controlled under various conditions to reach more than 84% in just 10 min, which indicates the high performance of the nanocatalyst along with the synergistic effect of ultrasonic irradiation. The structure of the products was identified by melting point as well as FTIR and ^1^H NMR spectroscopic methods. The Fe_3_O_4_@SiO_2_@PTS-APG nanocatalyst is easily prepared from commercially available, lower toxic and thermally stable precursors through a cost-effective, highly efficient and environmentally friendly procedure. The advantages of this method include simplicity of the operation, reaction under mild conditions, the use of an environmentally benign irradiation source, obtaining pure products with high efficiency in short reaction times without using a tedious path, which all of them address important green chemistry principles. Finally, a reasonable mechanism is proposed for the preparation of polyhydroquinoline (PHQ) and 1,4-dihydropyridine (1,4-DHP) derivatives in the presence of Fe_3_O_4_@SiO_2_@PTS-APG bifunctional magnetic nanocatalyst.

## Introduction

Recently, due to valuable advantages of the heterogeneous catalysts and the compatibility and conformity to green chemistry (GC) principles^1–6^, they have attracted the scientists’ attention for various organic transformations. One of the main factors in the reusability of these catalytic systems is their recyclability, which can be significantly improved by using magnetic materials such as Fe_3_O_4_, CuFe_2_O_4_, NiFe_2_O_4_ or similar compounds in the catalyst structure^5,7^. Indeed, magnetic materials lead to the easy and almost complete recovery of the corresponding heterogeneous catalytic systems^8–13^. However, to overcome the instability of magnetic Fe_3_O_4_ under environmental conditions and tendency to oxidation, silica is commonly utilized as a protective shell for the coating of the Fe_3_O_4_ magnetic nanoparticles (MNPs) to afford Fe_3_O_4_@SiO_2_ core-shell nanostructures ^14–18^. The obtained Fe_3_O_4_@SiO_2_ nanomaterial has several merits including prevention from agglomeration of Fe_3_O_4_ MNPs, increasing the catalyst activity via modification of silanol functional groups, high porosity of silica shell, nature benign, and cost-effectivity^19,20^. In recent years, various magnetic heterogeneous nanocomposites have been systematically investigated and reported, which are applied in different catalytic reactions ^21–27^. Furthermore, a variety of bio-based heterogeneous catalytic systems for application in different organic transformations have been reported as well^16,28–40^. Therefore, designing of new and efficient magnetic heterogeneous catalytic system based on naturally occurring materials including α-amino acids would be desirable.

Indeed, α-amino acids are one the most important groups of natural compounds that are vital for the synthesis of proteins in living cells. Several advantages of these compounds including bifunctionality, the presence of both NH_2_ and COOH groups simultaneously with proper geometry, optical activity (except glycine)^41^, natural abundance and cost-effectivity as well as ability for targeted modifications make them proper candidates for designing nontoxic and bio-based heterogeneous catalytic systems^42^. The prepared amino acid containing nanomaterials have been employed in different fields of chemical science including catalysts for organic synthesis, pharmaceuticals and food additives, medical industries, ionic liquids, CO_2_ sorbent, metal-organic frameworks (MOFs) and stabilizing of the selenium nanoparticles (SeNPs) used in cancer treatment ^43–52^. These characteristics and wide applications of amino acids encouraged our research team to use D-(–)-α-phenylglycine (APG) in the structure of novel nanomagnetic composite, which has promoted the synthesis of important *N*-containing six-membered heterocyclic rings.

Heterocycles belong to the largest and most diverse groups of organic compounds, which have found different chemical, medicinal, biomedical and industrial applications ^53–57^. One of the essential scaffolds of natural compounds such as vitamins, hormones, antibiotics, alkaloids and herbicides, numerous natural and synthetic biologically active drugs, agrochemicals and antivirals is heterocycles ^53,58,59^. Among different methods for the preparation of these bioactive compounds, multicomponent reactions (MCRs) strategy is one of the best pathways ^60–64^. MCRs have different advantages including formation of several chemical bonds during the reaction and synthesis of desired products in high efficiency, excellent selectivity, high atom economy in short reaction times and without the need for isolation or purification of the intermediates. As a result, there is no place for the formation of by-products and wastes in high quantities during such organic transformations. Accordingly, MCRs completely conform themselves to the GC principles ^65–70^.

**Figure 1:**
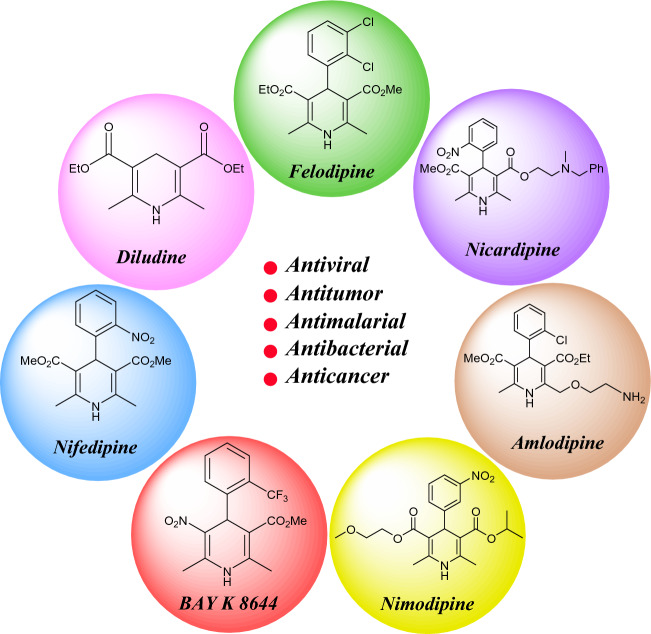
Some of the commercial biologically active 1,4-DHP derivatives.

**Figure 2:**
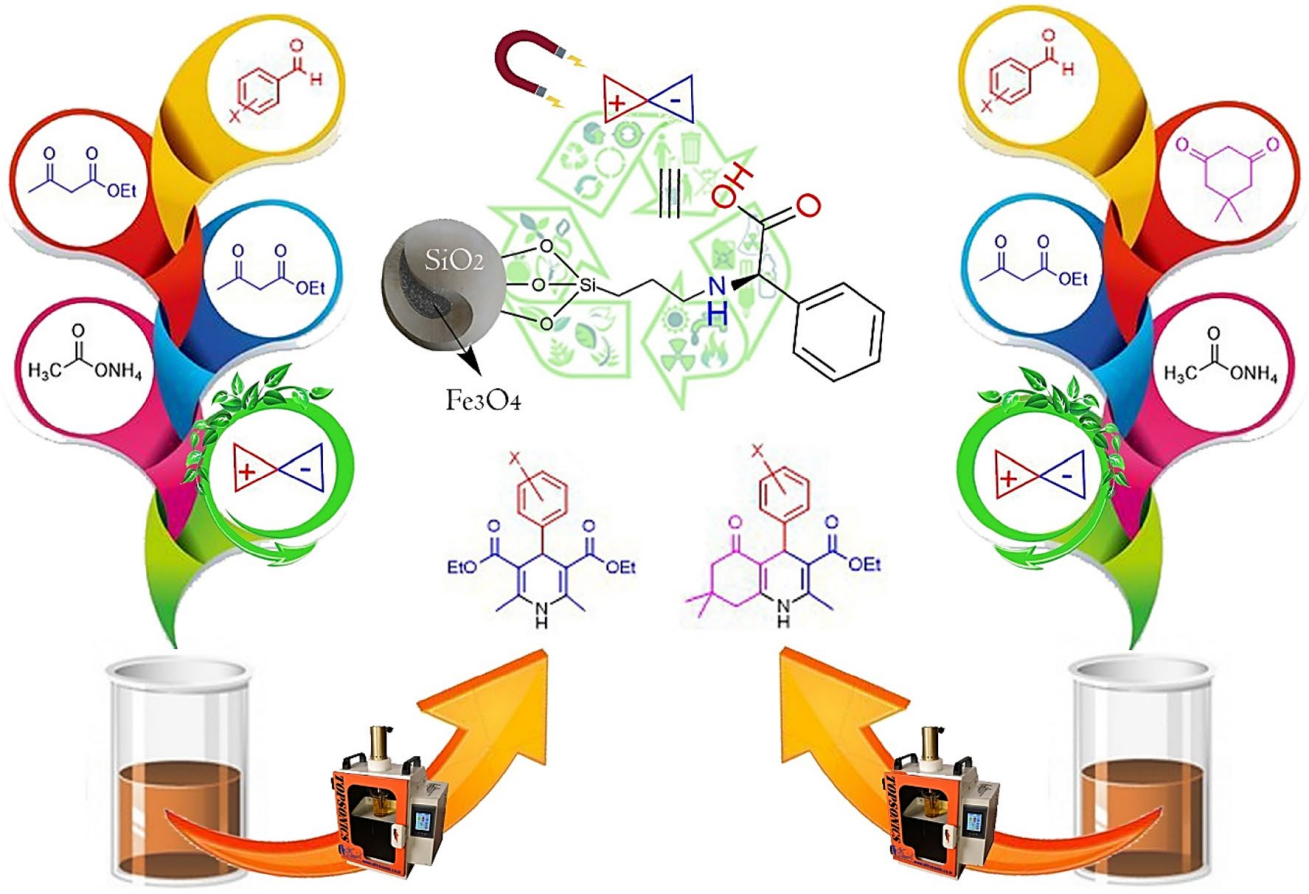
Synthesis of PHQ **6** and 1,4-DHP **7** derivatives catalyzed by the Fe_3_O_4_@SiO_2_@PTS-APG nanomagnetic catalyst (**1**).

1,4-Dihydropyridines (1,4-DHPs) and polyhydroquinolines (PHQs) are two useful products of the Hanztsch multicomponent reaction, which was introduced by Arthur Hanztsch in 1881. These compounds have attracted scientists’ attention and have found many applications in different areas of the medicinal chemistry including cardiovascular, antiviral, antitumor, antimalarial, antibacterial and anticancer compounds (Fig. 1) ^71–73^. Several methods and procedures have been developed for the synthesis of these important compounds including microwave irradiation^74^, solar thermochemical reactions^75^, and the use of various catalytic systems such as molecular iodine^76^, L-proline^77^, Fe_3_O_4_ magnetic nanoparticles^78^, ZnO nanoparticles^79^, polymers^80^ and HY-zeolite^81^. In spite of their merits, some of these methods suffer from disadvantages such as long reaction times, low yields, harsh conditions, high cost, the use of hazardous catalysts, toxic and volatile solvents, tedious workup, etc. Therefore, there is still room to design clean and green methodologies based on GC principles, especially by the use of heterogeneous catalytic systems as well as simultaneous use of with new energy inputs for chemical reactions including ultrasound^82,83^ and microwave irradiation^84–87^. In continuation of our ongoing researches in the field of application of heterogeneous multifunctional catalytic systems^4,17,18,28,29,69,70,88–93^ and ultrasound or microwave irradiation for different organic transformations^94–98^, we wish herein to report a new magnetic nanocomposite for the synthesis of bioactive Hanztsh 1,4-DHP and PHQ derivatives. The Fe_3_O_4_@SiO_2_@PTS-APG nanocatalyst was fabricated by preparing of the Fe_3_O_4_ central core, which was then coated by a SiO_2_ layer followed by introducing the D-(–)-α-phenylglycine, as a bifunctional organocatalyst moiety, through 3-chloropropyltrimethoxysilane (CPTES) linker. The as-prepared Fe_3_O_4_@SiO_2_@PTS-APG nanomagnetic catalytic system was examined properly in the synthesis of a wide range of PHQ **6** and 1,4-DHP **7** derivatives under ultrasonic or microwave irradiation in EtOH through MCR strategy (Fig. 2).

## Results and discussion

### Analysis and characterization of the magnetic core–shell catalyst functionalized with D-(–)-α-phenylglycine (Fe_3_O_4_@SiO_2_@PTS-APG, 1)

Different spectroscopic, microscopic and analytical techniques including Fourier transform infrared (FTIR) spectroscopy, field emission scanning electron microscopy (FESEM), vibrating sample magnetometer (VSM) analysis, X-ray powder diffraction (XRD) technique, energy-dispersive X-ray spectroscopy (EDX), and thermogravimetric analysis (TGA) were used to characterize the structure of core–shell magnetic nanocatalyst functionalized with D-(–)-α-phenylglycine (Fe_3_O_4_@SiO_2_@PTS-APG, **1**). The structure of Fe_3_O_4_@SiO_2_@PTS@APG has been illustrated in (Fig. 1S, Electronic Supplementary Information).

### FTIR spectra of the Fe_3_O_4_@SiO_2_@Pr-APG catalyst (1) and its components

**Figure 3:**
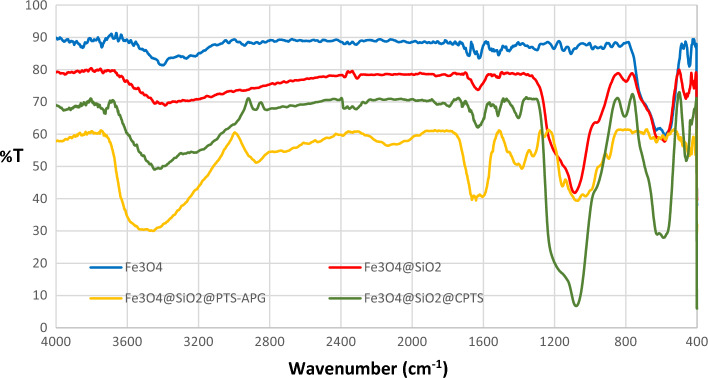
FTIR spectra of Fe_3_O_4_ (blue), Fe_3_O_4_@SiO_2_ (red), Fe_3_O_4_@SiO_2_@CPTS (green), Fe_3_O_4_@SiO_2_@PTS-APG (**1**, yellow).

The FTIR spectra of as-prepared Fe_3_O_4_@SiO_2_@PTS-APG nanomaterial (**1**) and its components containing inorganic and organic moieties have been shown in (Fig. 3). The band at 572 cm^−1^ in the spectrum of Fe_3_O_4_ (blue) is ascribed to the stretching vibration of Fe‒O‒Fe bonds, which is the representative of Fe_3_O_4_ nanoparticles structure. The absorbance bands at 1558 cm^−1^ and 3394 cm^−1^ are related to the bending and stretching vibrations of the OH groups on the surface of Fe_3_O_4_ nanoparticles, respectively. In the spectrum of Fe_3_O_4_@SiO_2_ (red), the absorption band at 440 cm^−1^ is associated to the bending vibration of the Si‒O‒Si functional groups, whereas the vibration peak at 800 cm^−1^ is related to the symmetric stretching vibration of the Si‒O‒Si groups. On the other hand, the asymmetric stretching absorption of the Si‒O‒Si groups appears at 1085 cm^−1^. These observations indicated successful fixation of the silica onto the surface of magnetite. After coating of the magnetic core by using silica, the introduction of linker can be deduced in the spectrum of Fe_3_O_4_@SiO_2_@PTS (green) form the strong absorption band at 588 cm^−1^, which showes the stretching vibration of the C‒Cl bond. The signal observed at 1070 cm^−1^ is attributed to the stretching vibration of the C‒O bond that has an overlap with the Si‒O‒Si asymmetric stretching vibrations. Finally, the spectrum of Fe_3_O_4_@SiO_2_@PTS-APG catalyst (yellow) shows that the catalyst has been functionalized with (D)-(–)-α-aminophenylacetic acid. Indeed, the stretching vibrations of the C‒H and C‒N are appeared at 2877 and 1382 cm^−1^, Furthermore, the absorption bands centered at 3490 and 1639 cm^−1^ are attributed to the acidic OH and carbonyl groups, respectively, which all confirm the structure of Fe_3_O_4_@SiO_2_@PTS-APG nanomaterial (**1**).

### EDS study of the Fe_3_O_4_@SiO_2_@PTS-APG catalyst (1)

**Figure 4:**
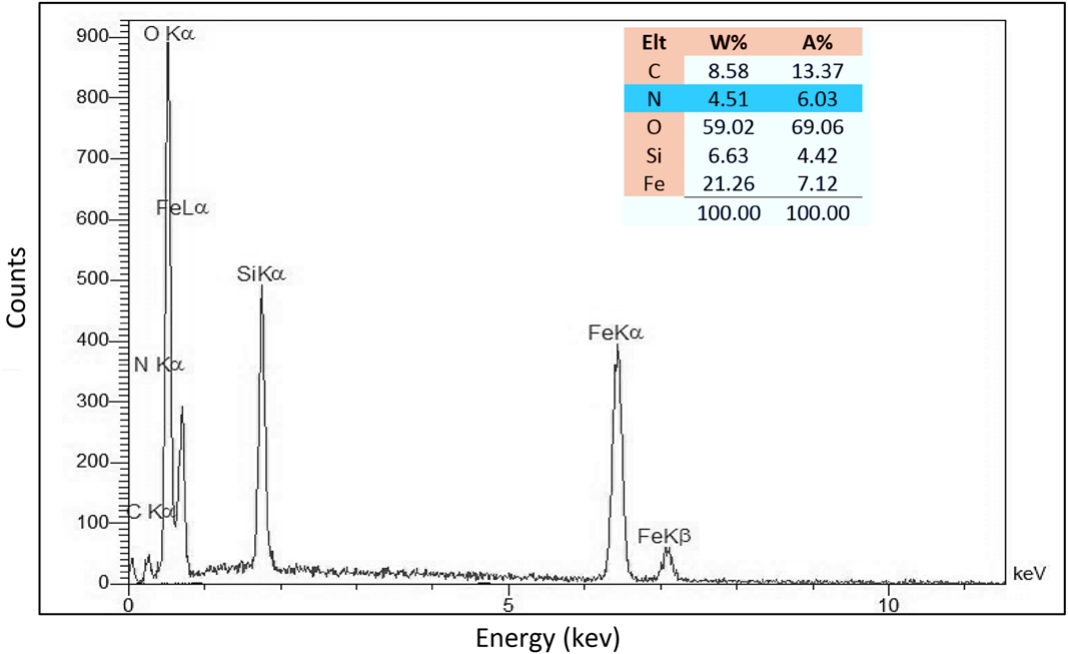
EDS of the Fe_3_O_4_@SiO_2_@PTS-APG catalyst (**1**).

The energy-dispersive X-ray spectroscopy (EDS) was also used to determine the composition of Fe_3_O_4_@SiO_2_@PTS-APG nanomaterial (**1**). The results are shown in Fig. 4. As can be seen, the catalyst contains C, N, O, Si and Fe elements. Moreover, the absence of the chlorine atom and the presence of the nitrogen atom indicate that the amino acid has been grafted covalently onto the surface of Fe_3_O_4_@SiO_2_@PTS magnetic core/shell nanoparticles and hence been stabilized.

### Microscopic imaging study of the Fe_3_O_4_@SiO_2_@PTS-APG catalyst (1)

**Figure 5:**
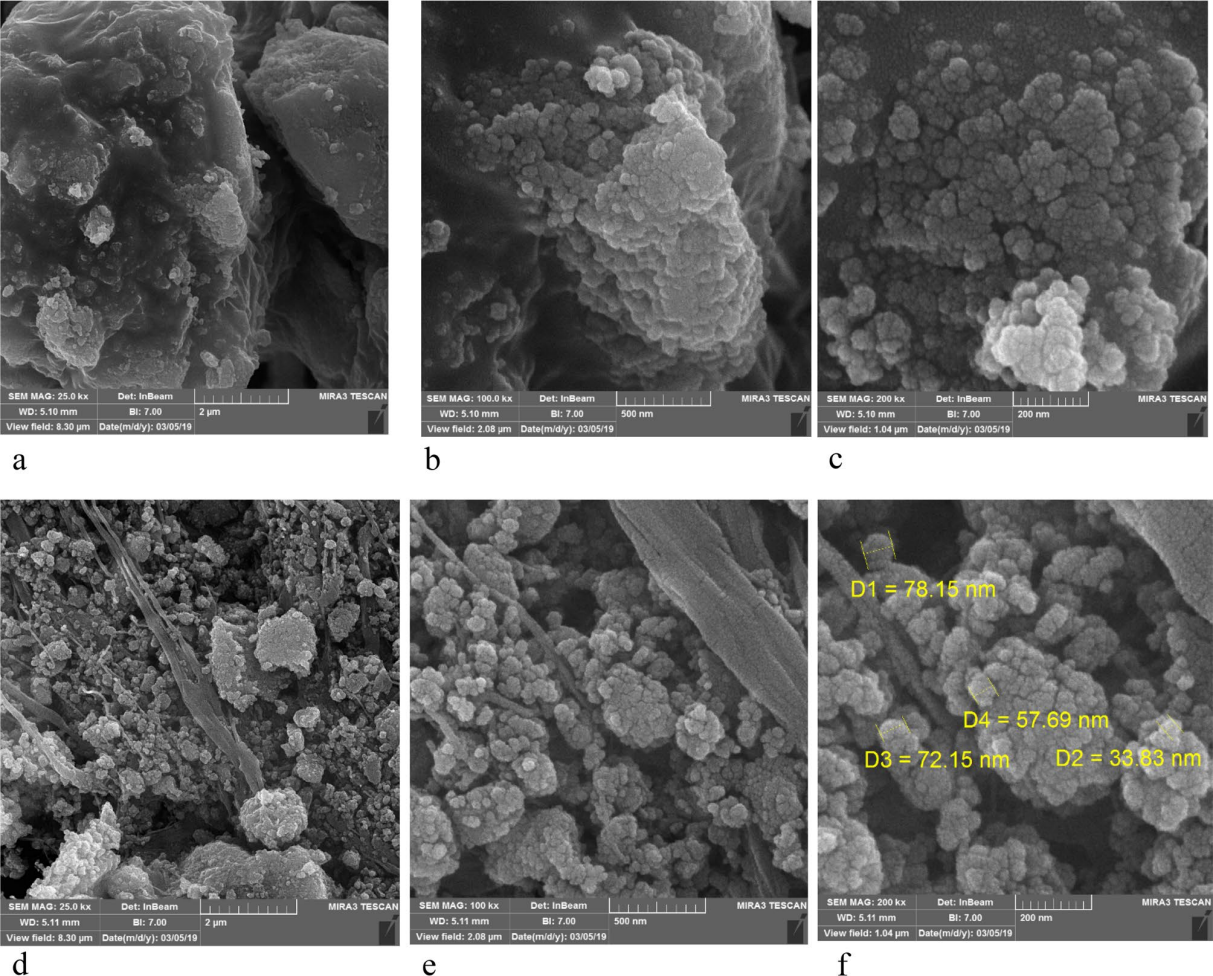
FESEM images of the Fe_3_O_4_@SiO_2_@PTS-APG nanomaterial (**1**).

In order to investigate the structure, morphological properties, and size of nanoparticles, the field emission scanning electron microscopy (FESEM) technique was employed. The FESEM images of the as-prepared nanocatalyst **1** are shown in (Fig. 5). The obtained images confirm the spherical morphology with non-smooth surface and proper-dispersion of the nanoparticles. Since the highly active areas of the catalyst are readily available, the surface area and the activity of nanocatalyst **1** were increased dramatically. According to Fig. 5f, it is clear that nanoparticles have a specific pattern and their average particle size is less than 80 nm.

### Vibrating sample magnetometer (VSM) analysis of the Fe_3_O_4_@SiO_2_@PTS-APG catalyst (1)

**Figure 6:**
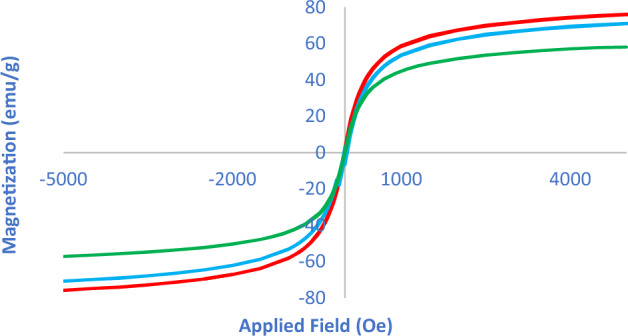
Magnetization curves of the Fe_3_O_4_ (red), Fe_3_O_4_@SiO_2_ (blue) and Fe_3_O_4_@SiO_2_@PTS**-**APG MNPs (**1**, green).

The magnetic properties of Fe_3_O_4_, Fe_3_O_4_@SiO_2_ and Fe_3_O_4_@SiO_2_@PTS-APG were determined by the vibrating sample magnetometer (VSM) technique at room temperature (Fig. 6). As can be seen, the magnetic values for Fe_3_O_4_, Fe_3_O_4_@SiO_2_, and Fe_3_O_4_@SiO_2_@PTS-APG are 75, 70, and 58 amu.g^−1^, respectively. The reduction in magnetic properties for Fe_3_O_4_@SiO_2_ and Fe_3_O_4_@SiO_2_@PTS-APG compared to the bare Fe_3_O_4,_ confirms formation of a thin layer of silica, surface modification with propylene trialkoxysilane, and introduction of the D-(–)-α-phenylglycine in the last stage.

### XRD study of the Fe_3_O_4_@SiO_2_@PTS-APG catalyst (1)

**Figure 7:**
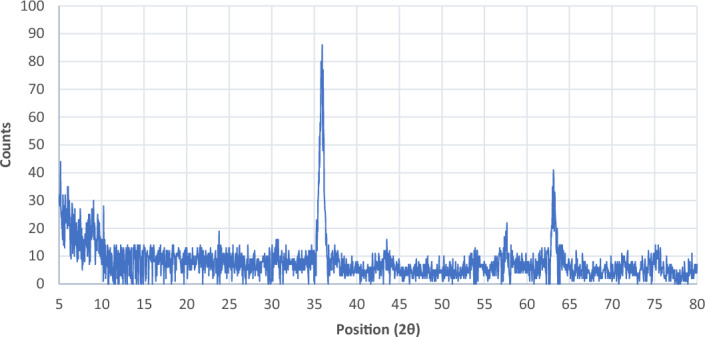
XRD Pattern of the Fe_3_O_4_@SiO_2_@PTS-APG nanocatalyst (**1**).

The X-ray diffraction** (**XRD) pattern of the Fe_3_O_4_@SiO_2_@PTS-APG nanoparticles **1** is shown in (Fig. 7). The structure of the as-prepared catalyst is fully compatible with the standard patterns of Fe_3_O_4_ (card. No JCPDS, 01-088-0315), Fe_3_O_4_@SiO_2_ (card. No JCPDS, 01–082-1572) and D-(–)-α-phenylglycine (card. No JCPDS, 00-013-0988). The diffraction signals (2θ) at 25, 28, and 31° correspond to the D-(–)-α-phenylglycine, which confirms its stabilization onto the surface of silica-coated magnetic nanoparticles.

### Thermogravimetric analysis (TGA) of the Fe_3_O_4_@SiO_2_@PTS-APG catalyst (1)

**Figure 8:**
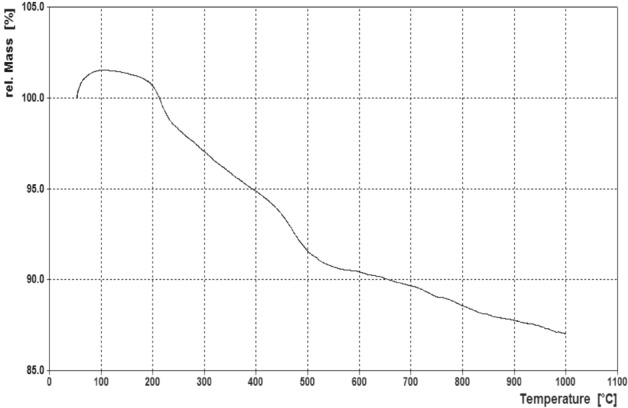
TGA curve of the Fe_3_O_4_@SiO_2_@PTS-APG nanomaterial (**1**).

In order to investigate the thermal stability of the Fe_3_O_4_@SiO_2_@PTS-APG hybrid organosilica nanocatalyst (**1**), its thermogravimetric analysis (TGA) was performed under N_2_ atmosphere at the range of 50 to 1000 °C. The total weight loss of the nanocatalyst was about 14% (Fig. 8). As can be seen, by a gradual increase in the temperature to 95 °C, a slight increase in the weight of the nanocatalyst was observed, which may be due to the absorption of moisture by its hygroscopic surface. The first weight loss started at 100 °C is related to the removal of water or residual organic solvents in the nanocatalyst. At higher temperatures at about 250 – 450 °C as well as 450 – 600 °C, the pure organic component and the organosilica coating are decomposed, respectively. Finally, after 600 °C, a gradual decrease in weight is observed, which is related to dehydration of both SiO_2_ and magnetic components.

### Optimization of the Hantzsch reaction catalyzed by the Fe_3_O_4_@SiO_2_@PTS-APG nanomaterial (1)

In order to optimize the Hantzsch reaction conditions for the synthesis of polyhydroquinoline derivatives (PHQs) in the presence of Fe_3_O_4_@SiO_2_@PTS-APG nanomaterial (**1**), the one-pot four-component reaction of 4-(dimethylamino)benzaldehyde (**2a**), ammonium acetate (**3**), ethyl acetoacetate (**4**), dimedone (**5**) was selected as a model reaction. Thus, to improve the synthesis of polyhydroquinoline derivatives and choose the best reaction conditions, a systematic study was accomplished by considering different parameters and variants including solvents and catalyst loading as well as ultrasonic (US) or microwave (MW) irradiation and classical heating energy inputs, and reaction time. The results of this part of our study are summarized in Table 1. As shown in Table 1, the model reaction in the absence of the catalyst **1** afforded no significant yield (entry 1). However, in the presence of the catalyst **1** and in various organic solvents, the desired product ethyl 4-(4-(dimethylamino)phenyl)-2,7,7-trimethyl-5-oxo-1,4,5,6,7,8-hexahydroquinoline-3-carboxylate (**6a**) was formed in higher yields (entries 2–6). Indeed, the best result was observed in EtOH 96% as a protic polar solvent (Table 1, entry 2).

**Table 1:**
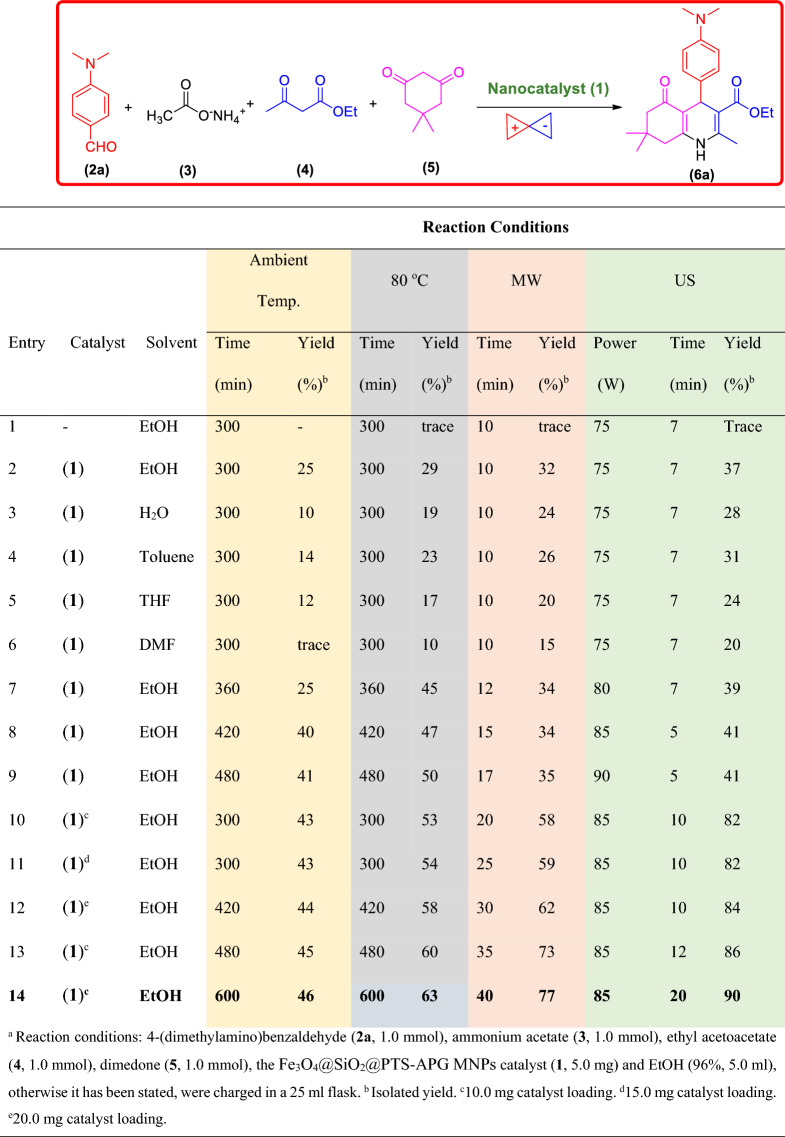
Optimization of the reaction conditions for the synthesis of **6a** in the presence of Fe_3_O_4_@SiO_2_@PTS-APG nanomaterial (**1**)^a^.

^a^Reaction conditions: 4-(dimethylamino) benzaldehyde (**2a**, 1.0 mmol), ammonium acetate (**3**, 1.0 mmol), ethyl acetoacetate (**4**, 1.0 mmol), dimedone (**5**, 1.0 mmol), the Fe_3_O_4_@SiO_2_@PTS-APG MNPs catalyst (**1**, 5.0 mg) and EtOH (96%, 5.0 ml), otherwise it has been stated, were charged in a 25 ml flask. ^b^Isolated yield. ^c^10 mg catalyst loading. ^d^15.0 mg catalyst loading. ^e^20.0 mg catalyst loading.

After finding of the appropriate solvent, screening of different ultrasonic or microwave irradiation powers was investigated. Generally, higher yields of the desired product **6a** were obtained under ultrasonic or microwave irradiation compared to the classical heating. Furthermore, it was observed that by increasing the ultrasonic irradiation power from 80 to 85 W the yield is increased and the reaction time is decreased (Table 1, entries 7 and 8). However, with an increase in ultrasonic irradiation power from 85 to 90 W, the yield and the reaction time remained constant (Table 1, entry 9). These results indicated that 85 W for ultrasonic irradiation is the optimal irradiation power. Finally, the best result was observed by using the effective amount of the catalyst **1** (10.0 mg) and increasing the reaction time to 20 min (Table 1**,** entry 14). In general, the best conditions for the synthesis of **6a** was using 10.0 mg of catalyst in the EtOH, as a green solvent, for 20 min under 85 W ultrasonic irradiation power (Table 1, entry 14).

After optimizing of the reaction conditions, several PHQs **6a**-**j** were synthesized under optimal conditions, and the results are summarized in Table 2. As shown in Table 2, substituted aldehydes containing electron-donating or electron-withdrawing groups survived under optimized reaction conditions to afford high to excellent yields of their desired products.

**Table 2:**
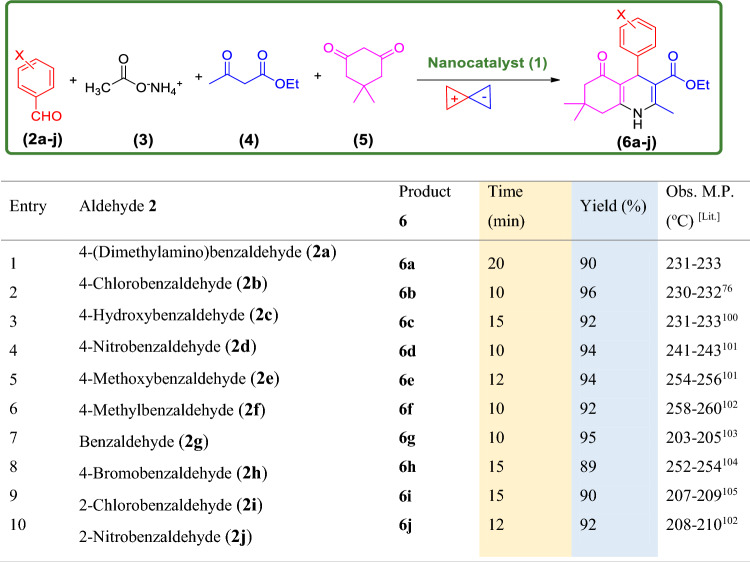
Synthesis of different polyhydroquinoline derivatives using Fe_3_O_4_@SiO_2_@PTS-APG NPs catalyst (**1**) under optimized reaction conditions^a^.

^a^Reaction conditions: aldehyde derivatives (**2a-j**, 1.0 mmol), ammonium acetate (**3**, 1.0 mmol), ethyl acetoacetate (**4**, 1.0 mmol), dimedone (**5**, 1.0 mmol), and catalyst (**1**, 10 mg) in EtOH (96%, 3.0 ml) under ultrasonic irradiation.

By considering the obtained satisfactory results in the synthesis of PHQs **6a-j**, the use of nanomagnetic catalyst **1** for the synthesis of 1,4-DHPs was investigated under the optimized conditions. In this part of our study, the reaction of 4-chlorobenzaldehyde (**2b**), ammonium acetate (**3**), ethyl acetoacetate (**4**) promoted by the Fe_3_O_4_@SiO_2_@PTS-APG nanomaterial (**1**) was investigated, as the model reaction, for the synthesis of desired product **7a**. The results are summarized in Table 3. The best result was obtained by using 10.0 mg of the catalyst **1** in EtOH under ultrasonic irradiation (Table 3, entry 6). Accordingly, several 1,4-DHPs were synthesized and the obtained results are summarized in Table 4.

**Table 3:**
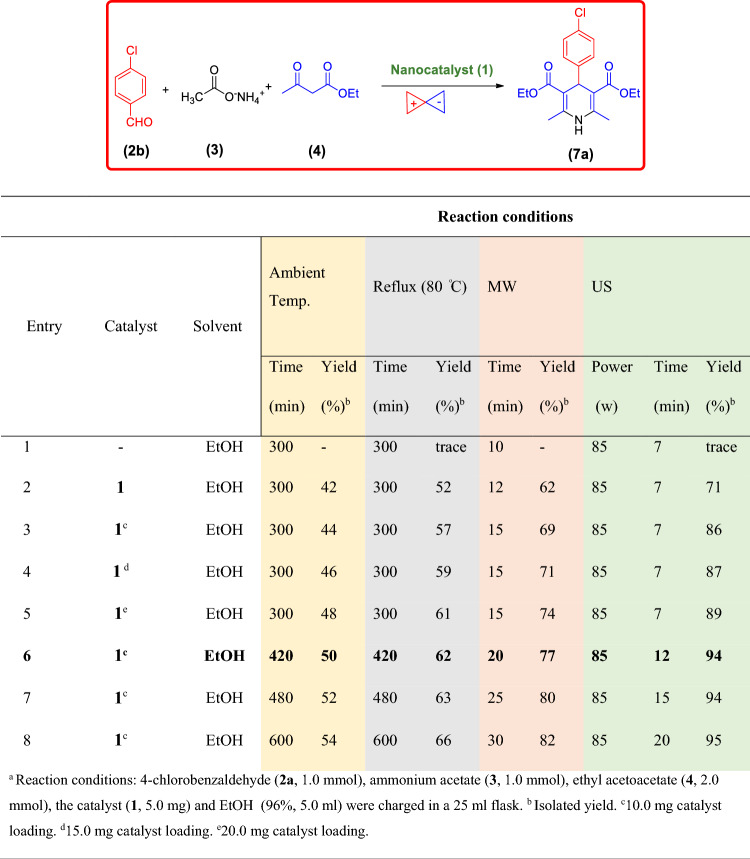
Optimization of the reaction conditions for the synthesis of **7a** in the presence of Fe_3_O_4_@SiO_2_@PTS-APG nanocatalyst (**1**)^a^.

^a^Reaction conditions: 4-chlorobenzaldehyde (**2a**, 1.0 mmol), ammonium acetate (**3**, 1.0 mmol), ethyl acetoacetate (**4**, 2.0 mmol), the catalyst (**1**, 5.0 mg) and EtOH (96%, 5.0 ml) were charged in a 25 ml flask. ^b^Isolated yield. ^c^10.0 mg catalyst loading. ^d^15.0 mg catalyst loading. ^e^20.0 mg catalyst loading.

**Table 4:** Synthesis of different 1,4-DHPs using the Fe_3_O_4_@SiO_2_@PTS-APG MNPs catalyst (**1**)^a^.


Entry	Aldehyde **2**	Product **7**	Time (min)	Yield (%)	Obs. M.P. (^o^C) ^[Lit.]^
1	4-Chlorobenzaldehyde (**2b**)	**7a**	12	94	142–144^105^
2	4-Nitrobenzaldehyde (**2d**)	**7b**	10	90	129–131^105^
3	3-Nitrobenzaldehyde (**2k**)	**7c**	10	87	162–164^106^
4	Benzaldehyde (**2g**)	**7d**	12	88	156–158^107^
5	4-Methylbenzaldehyde (**2f**)	**7e**	17	85	137–139^106^
6	4-Bromobenzaldehyde (**2h**)	**7f.**	12	90	162–164^107^
7	4-Hydroxybenzaldehyde (**2c**)	**7 g**	10	90	200–202^108^
8	2,4-Dichlorobenzaldehyde (**2l**)	**7 h**	20	87	188–190^105^
9	4-Methoxybenzaldehyde (**2e**)	**7i**	15	90	158–160^71^
10	Furan-2-carbaldehyde (**2m**)	**7j**	12	88	160–162^71^
11	Butyraldehyde (**2n**)	**7 k**	12	84	121–123^109^

^a^Reaction conditions: aldehyde derivatives (**2a**-**j**, 1.0 mmol), ammonium acetate (**3**, 1.0 mmol), ethyl acetoacetate (**4**, 2.0 mmol) and catalyst (10.0 mg) in EtOH (96%, 3.0 ml) under ultrasonic irradiation.

### Mechanism of the Hantzsch reaction catalyzed by the Fe_3_O_4_@SiO_2_@PTS-APG nanocatalyst (1)

**Figure 9:**
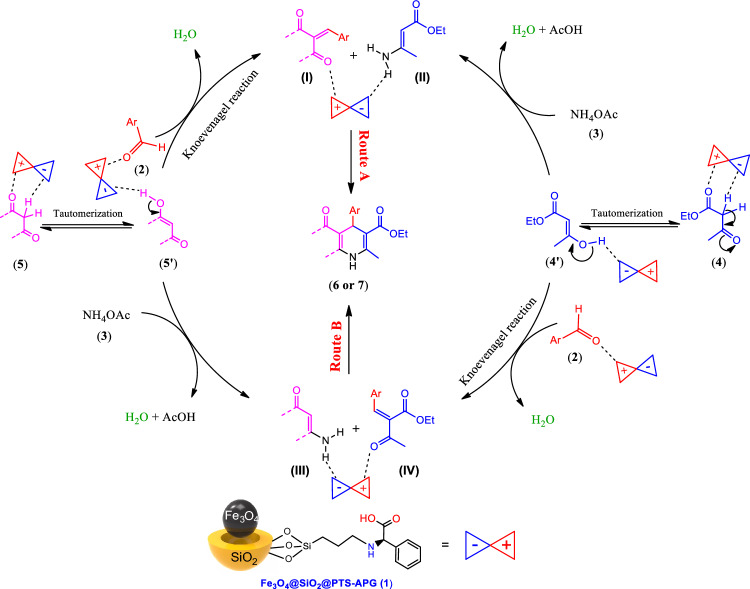
Proposed mechanism for the synthesis of polyhydroquinoline **6** or 1,4-dihydropyridine **7** derivatives catalyzed by the Fe_3_O_4_@SiO_2_@PTS-APG MNPs (**1**).

According to the obtained results and based on the bifunctional structure of the catalyst **1** that contains both acidic and basic sites on the MNPs as well as literature survey^4,16,31,70,110–114^, a rational mechanism for the formation of polyhydroquinoline **6** or 1,4-dihydropyridine **7** derivatives in the presence of Fe_3_O_4_@SiO_2_@PTS-APG nanocatalyst has been proposed through the Hantzch MCR (Fig. 9). Accordingly, these compounds can be synthesized through two different routes **A** or **B** and in several steps. Based on route **A**, the acidic and basic sites of the catalyst **1** activates dimedone **5** to increase its enol form concentration for subsequent reacting with the activated carbonyl functional group of aldehydes **2** to afford intermediate (**I**) via Knoevenagel condensation. On the other side of the catalytic cycle, the reaction between the enol form of β-ketoester **4** activated by the nanocatalyst **1** with NH_4_OAc (**3**) produces enamine (**II**). Subsequently, the bifunctional catalyst **1** activates both intermediates (**I**) and (**II**) to participate in the catalyzed Michael addition followed by cyclization for the synthesis of final polyhydroquinoline **6** or 1,4-dihydropyridine **7** derivatives. Indeed, route **B** is generally similar to route **A**. However, it differs from route **A** by considering the sequence of reacting of the used 1,3-dicarbonyl **4** or **5** with the activated carbonyl functional group of aldehydes **2** or NH_4_OAc (**3**), which produce intermediates (**IV**) and (**III**), respectively. Finally, Michael addition of intermediates (**III**) and (**IV**) followed by cyclization both promoted by the Fe_3_O_4_@SiO_2_@PTS-APG to afford polyhydroquinoline **6** or 1,4-dihydropyridine **7** derivatives.

### Comparison of the catalytic activity of Fe_3_O_4_@SiO_2_@PTS-APG NPs 1 with other catalytic systems

To compare the performance and activity of the Fe_3_O_4_@SiO_2_@PTS-APG NPs (**1**) with other previously reported catalysts, several products among the PHQs **6** and 1,4-DHPs **7** were selected and the obtained results were evaluated with other previous methods. The results have been summarized in Table 5. As can be implied from Table 5, the as-prepared nanocomposite **1** shows the better results in terms of catalyst loading, obtained yields and reaction time than other catalysts listed in the Table. In summary, the simultaneous use of the nanocatalyst **1** and ultrasonic irradiation demonstrates several advantages including excellent yields, high selectivity, short reaction time, and mild reaction conditions.

**Table 5:**
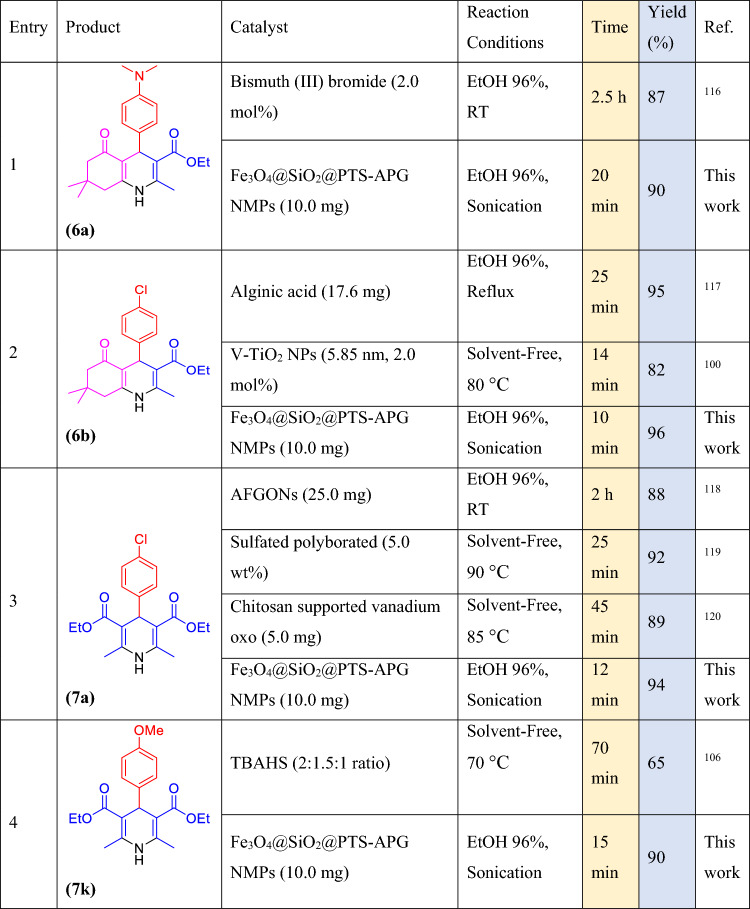
Comparison of the efficiency of the Fe_3_O_4_@SiO_2_@PTS-APG MNPs (**1**) with other reported catalytic systems for the synthesis of some PHQ 6 and 1,4-DHP 7 derivatives.

### Reusability of the Fe_3_O_4_@SiO_2_@PTS-APG NPs (1) for the Hantzsch synthesis of PHQs 6 and 1,4-DHPs 7

**Figure 10:**
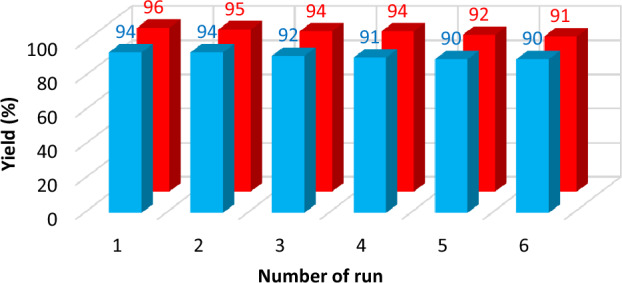
Reusability of the Fe_3_O_4_@SiO_2_@PTS-APG MNPs (**1**) in the synthesis of **6b** (red) and **7a** (blue) under optimized conditions.

The recovery of heterogeneous catalysts in the chemical reactions is one of the most important factors in their evaluation and the application in the industrial sectors as well. To investigate the reusability of the catalyst, it was separated from the reaction mixture and washed with EtOH 96% after each run. Then, the recycled catalyst was dried in an oven at 70 °C for 2 h. The recovered nanocomposite (**1**) was reused for subsequent experiments up to five times under the same reaction conditions. The reusability of the Fe_3_O_4_@SiO_2_@PTS-APG NPs (**1**) was examined in the synthesis of products **6b** and **7a** under optimized reaction conditions. It is generally accepted that there are three fundamental reasons for catalyst deactivation, i.e. poisoning, coking, or fouling and ageing^120^. In the case of our catalyst, a combination of these phenomena may be considered as the main reasons for decreasing of the catalytic activity. According to the obtained results in Fig. 10, it can be concluded that this heterogeneous catalyst can be used at least six times without significant loss in its catalytic activity.

## Experimental

### Materials and methods

All the chemicals and solvents were purchased from Merck and used without further purification, except for benzaldehyde and furfural, which were used as fresh distilled samples. The progress of reactions, as well as the purity of products, were checked using F254 silica-gel pre-coated TLC plates with n-hexane and ethyl acetate (1:1, v/v) as eluent. The melting points were determined on a Buchi melting point apparatus and are uncorrected. FTIR spectra were recorded on a Perkin Elmer FTIR spectrophotometer using KBr pellets in the range of 399–4490 cm^−1^. ^1^H NMR spectra were recorded on a Bruker 500 MHz for samples in CDCl_3_, as the solvent, at ambient temperature. Ultrasonication was performed in a BANDELIN ultrasonic HD 3200 instrument with probe model US 70/T with a diameter of 6 mm that was immersed directly into the reaction mixture. A National microwave oven, model no. NN-K571MF (1000 W), was used for microwave-assisted reactions. Scanning electron microscopy (SEM) images were obtained on an MIRA3 TESCAN instrument operated at 30 kV accelerating voltage. Magnetization measurements were carried out on a BHV-55 vibrating sample magnetometer (VSM). Thermogravimetric analysis (TGA) was recorded utilizing a Bahr company STA 504 instrument. Energy-dispersive X-ray (EDX) analysis was accomplished by a FESEM-SIGM (German) instrument.

### Preparation of the magnetite (Fe_3_O_4_) NPs coated with silica (Fe_3_O_4_@SiO_2_)

FeCl_3_.6H_2_O (4.82 g) and FeCl_2_.4H_2_O (2.25 g) were dissolved in 40 ml deionized water at 80 °C for 20 min under nitrogen atmosphere and vigorous stirring. Then, aqueous NH_3_ (25%, 10 ml) was added into the solution and stirred vigorously at 70 °C for 1 h. The color of the bulk solution turned from orange to black immediately. Then, the precipitated Fe_3_O_4_ nanoparticles were separated from the mixture using an external magnet, washed several times with deionized water and EtOH 96% until reaching to the neutral pH, and left to dry in the air for 4 h. Afterward, Fe_3_O_4_ NPs (1.0 g) was dispersed EtOH (96%, 40 ml) and deionized water (15 ml) by ultrasonic irradiation in a bath for 20 min. After that, TEOS (1.2 ml) was added to the mixture and sonicated for 15 min. Finally, aqueous ammonia (25%, 1.2 ml) was added gradually under mechanical stirring at 30 °C. After 12 h, the silica-coated magnetic nanoparticles were filtered, washed several times with EtOH 96% and distilled water, and dried at 50 °C for 6 h (Fig. 1S, Electronic Supplementary Information).

### Preparation of the modified chloropropyl silica-coated magnetite NPs (Fe_3_O_4_@SiO_2_@CPTS)

Fe_3_O_4_@SiO_2_ nanoparticles (1.0 g) were suspended in toluene (40 ml) using sonication. Then, they were functionalized by using chloropropyltriethoxysilane (CPTS, 1.0 ml) followed by reflux for 24 h under nitrogen atmosphere. The obtained precipitated solid was collected and washed several times with EtOH 96% and finally dried at 80 °C to afford desired Fe_3_O_4_@SiO_2_@CPTS NPs (Fig. 1S, Electronic Supplementary Information).

### Immobilization of the D-(−)-α-phenylglycine (APG) on the surface of modified silica-coated magnetite NPs

Fe_3_O_4_@SiO_2_@CPTS Powder (1.0 g) was suspended in EtOH (96%, 40 ml). Then, D-(−)-α-phenylglycine (1.0 g) was added into the mixture and refluxed at 70 °C for 24 h. The produced solid was separated using a magnet and washed several times with EtOH 96%. Finally, the obtained sample was dried under vacuum at 80 °C for 24 h to afford Fe_3_O_4_@SiO_2_@PTS-APG NPs (**1**) (Fig. 1S, Electronic Supplementary Information).

### General procedure for the synthesis of PHQs 6 and 1,4-DHPs 7 catalyzed by the Fe_3_O_4_@SiO_2_@PTS-APG NPs (1)

A mixture of aldehyde derivatives (**2**, 1.0 mmol), ammonium acetate (**3**, 1.0 mmol), ethyl acetoacetate (**4**, 1.0 mmol), dimedone (**5**, 1.0 mmol), the catalyst (**1**, 10.0 mg) and EtOH (96%, 5.0 ml) were charged into a round-bottom flask and the obtained mixture was irradiated by an ultrasonic probe under mentioned conditions in Table 2. The formation of the products **6** was monitored by TLC. In order to synthesis of 1,4-DHP derivatives **7**, the catalyst (**1**, 10.0 mg), aldehyde derivatives (**2**, 1.0 mmol), ammonium acetate (**3**, 1.0 mmol), ethyl acetoacetate (**4**, 2.0 mmol) and EtOH (96%, 2.0 ml) were added into a round-bottom flask and then irradiated by using an ultrasonic probe under mentioned conditions in Table 4. The progress of the reaction was monitored by TLC. After completion of the Hantzsch reaction in each case, the catalyst was removed by an external magnet after adding EtOH 96% for complete dissolution of the products under heating. Then, the pure products **6** or **7** were obtained by recrystallization of the crude reaction mixture from EtOH H_2_O. The chemical structure of the known compounds was confirmed by comparing their melting points, FTIR, and ^1^H NMR spectra (Figs. 10S–21S, Electronic Supplementary Information) with the reported data in the literature. The physical and spectral information of compounds **6a** and **7a** are given in Table 6.

**Table 6:**
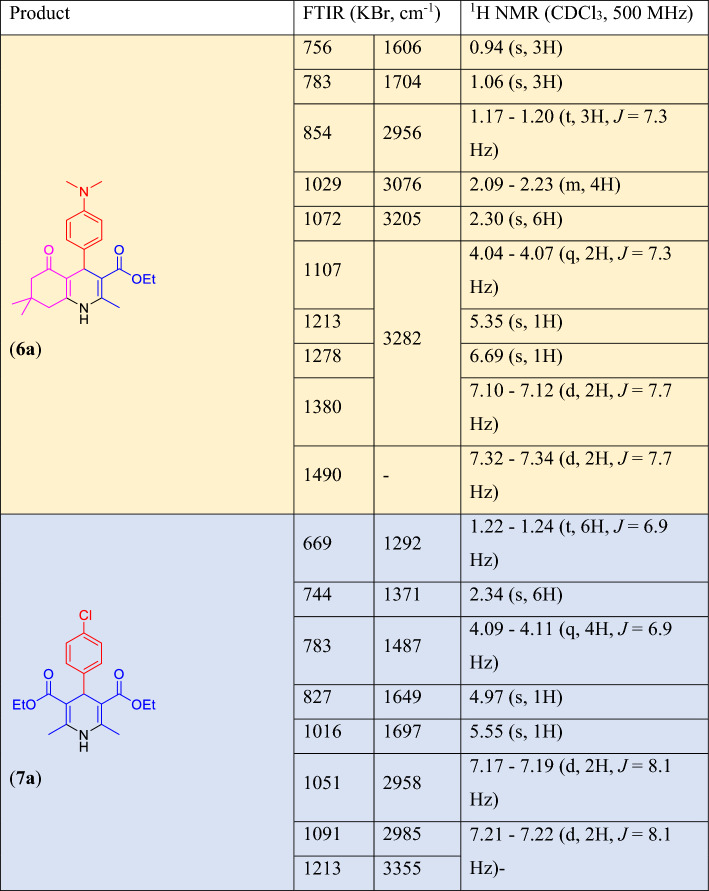
FTIR and ^1^H NMR spectral data of compounds **6a** and **7a.**

## Conclusion

In this work, we have developed a robust and efficient bifunctional organocatalyst immobilized on the surface of modified silica-coated magnetite (Fe_3_O_4_@SiO_2_@PTS-APG) NPs. The Fe_3_O_4_@SiO_2_@PTS-APG nanomagnetic catalyst was employed successfully for the synthesis of different polyhydroquinoline (PHQ) and 1,4-dihydropyridine (1,4-DHP) derivatives through the Hantzsch multicomponent reaction in EtOH as a green solvent. Various energy sources were used for the synthesis of Hantzsch ester derivatives, among which ultrasonic demonstrated the best efficiency. Indeed, ultrasonic irradiation demonstrating a synergistic effect with Fe_3_O_4_@SiO_2_@PTS-APG nanocatalyst accelerate the reaction rate. This new protocol has significant advantages compared to other commonly used methods including avoiding the use of harmful solvents, high efficiency, short reaction times, environmentally friendly, and cost-effectiveness. In addition, the prepared heterogeneous nanocatalyst demonstrates good recycling capability and it was easily recycled and reused at least five times without significant loss in its catalytic activity. Accordingly, the principles of GC were covered by the use of a recyclable catalyst, green solvent, and efficient energy source, all of which are environmentally benign.

## Supplementary Information

Supplementary Information.

## Data Availability

All data generated or analyzed during this study are included in this published article [and its supplementary information files].

## References

[1] Wu H, Wang Z, Tao L (2017). The Hantzsch reaction in polymer chemistry: Synthesis and tentative application. Polym. Chem..

[2] Zhang Q (2016). Lighting up the PEGylation agents via the Hantzsch reaction. Polym. Chem..

[3] Rezaie M, Dinari M, Chermahini AN, Saraji M, Shahvar A (2020). Preparation of kapa carrageenan-based acidic heterogeneous catalyst for conversion of sugars to high-value added materials. Int. J. Biol. Macromol..

[4] Sam M, Dekamin MG, Alirezvani Z (2021). Dendrons containing boric acid and 1,3,5-tris (2-hydroxyethyl) isocyanurate covalently attached to silica-coated magnetite for the expeditious synthesis of Hantzsch esters. Sci. Rep..

[5] Veisi H (2021). Bio-inspired synthesis of palladium nanoparticles fabricated magnetic Fe_3_O_4_ nanocomposite over Fritillaria imperialis flower extract as an efficient recyclable catalyst for the reduction of nitroarenes. Sci. Rep..

[6] Chen X (2017). Sixteen isostructural phosphonate metal–organic frameworks with controlled Lewis acidity and chemical stability for asymmetric catalysis. Nat. Commun..

[7] Alirezvani Z, Dekamin MG, Valiey E (2019). Cu (II) and magnetite nanoparticles decorated melamine-functionalized chitosan: A synergistic multifunctional catalyst for sustainable cascade oxidation of benzyl alcohols/Knoevenagel condensation. Sci. Rep..

[8] Ghomi JS, Zahedi S (2017). Novel ionic liquid supported on Fe_3_O_4_ nanoparticles and its application as a catalyst in Mannich reaction under ultrasonic irradiation. Ultrason. Sonochem..

[9] Mak CA, Pericas MA, Fagadar-Cosma E (2018). Functionalization of A3B-type porphyrin with Fe_3_O_4_ MNPs. Supramolecular assemblies, gas sensor and catalytic applications. Catal. Today.

[10] Neamtu M (2018). Functionalized magnetic nanoparticles: Synthesis, characterization, catalytic application and assessment of toxicity. Sci. Rep..

[11] Zhou Y (2017). Synchronized purification and immobilization of his-tagged β-glucosidase via Fe_3_O_4_/PMG core/shell magnetic nanoparticles. Sci. Rep..

[12] Alnadari F (2020). Immobilization of β-glucosidase from *Thermatoga*
*maritima* on chitin-functionalized magnetic nanoparticle via a novel thermostable chitin-binding domain. Sci. Rep..

[13] Li Q (2017). Correlation between particle size/domain structure and magnetic properties of highly crystalline Fe_3_O_4_ nanoparticles. Sci. Rep..

[14] Ranganath KV, Glorius F (2011). Superparamagnetic nanoparticles for asymmetric catalysis—A perfect match. Catal. Sci. Technol..

[15] Karami S, Dekamin MG, Valiey E, Shakib P (2020). DABA MNPs: a new and efficient magnetic bifunctional nanocatalyst for green synthesis of biologically active pyrano [2, 3-c] pyrazole and benzylpyrazolyl coumarin derivatives. New J. Chem..

[16] Rostami N, Dekamin MG, Valiey E (2023). Chitosan-EDTA-Cellulose bio-based network: A recyclable multifunctional organocatalyst for green and expeditious synthesis of Hantzsch esters. Carbohydr. Polym. Technol. Appl..

[17] Safapoor S, Dekamin MG, Akbari A, Naimi-Jamal MR (2022). Synthesis of (E)-2-(1H-tetrazole-5-yl)-3-phenylacrylenenitrile derivatives catalyzed by new ZnO nanoparticles embedded in a thermally stable magnetic periodic mesoporous organosilica under green conditions. Sci. Rep..

[18] Ishani M, Dekamin MG, Alirezvani Z (2018). Superparamagnetic silica core-shell hybrid attached to graphene oxide as a promising recoverable catalyst for expeditious synthesis of TMS-protected cyanohydrins. J. Colloid Interface Sci..

[19] Shao M (2012). Preparation of Fe_3_O_4_@SiO_2_@ layered double hydroxide core–shell microspheres for magnetic separation of proteins. J. Am. Chem. Soc..

[20] Wang P, Liu H, Niu J, Li R, Ma J (2014). Entangled Pd complexes over Fe_3_O_4_@SiO_2_ as supported catalysts for hydrogenation and Suzuki reactions. Catal. Sci. Technol..

[21] Liu J (2018). Fe/Beta@SBA-15 core-shell catalyst: Interface stable effect and propene poisoning resistance for no abatement. AIChE J..

[22] Jia L (2022). Interface engineering of a bifunctional Cu-SSZ-13@CZO core-shell catalyst for boosting potassium Ion and SO_2_ tolerance. ACS Catal..

[23] Liu J (2020). Deep understanding of strong metal interface confinement: A journey of Pd/FeOx catalysts. ACS Catal..

[24] Dohendou M, Pakzad K, Nezafat Z, Nasrollahzadeh M, Dekamin MG (2021). Progresses in chitin, chitosan, starch, cellulose, pectin, alginate, gelatin and gum based (nano) catalysts for the Heck coupling reactions: A review. Int. J. Biol. Macromol..

[25] Jabbar ZH, Ammar SH, Ebrahim SE (2021). Enhanced visible-light photocatalytic bacterial inhibition using recyclable magnetic heterogeneous nanocomposites (Fe_3_O_4_@SiO_2_@Ag_2_WO_4_@Ag_2_S) in core/shell structure. Environ. Nanotechnol. Monit. Manag..

[26] Gawande MB, Monga Y, Zboril R, Sharma R (2015). Silica-decorated magnetic nanocomposites for catalytic applications. Coord. Chem. Rev..

[27] Wang J (2023). Polymer-based nanocomposites: Role of interface for effective microwave absorption. Mater. Today Phys..

[28] Valiey E, Dekamin MG, Bondarian S (2023). Sulfamic acid grafted to cross-linked chitosan by dendritic units: A bio-based, highly efficient and heterogeneous organocatalyst for green synthesis of 2,3-dihydroquinazoline derivatives. RSC Adv..

[29] Valiey E, Dekamin MG (2022). Supported copper on a diamide–diacid-bridged PMO: An efficient hybrid catalyst for the cascade oxidation of benzyl alcohols/Knoevenagel condensation. RSC Adv..

[30] Rostami N, Dekamin M, Valiey E, Fanimoghadam H (2022). Chitosan-EDTA-Cellulose network as a green, recyclable and multifunctional biopolymeric organocatalyst for the one-pot synthesis of 2-amino-4H-pyran derivatives. Sci. Rep..

[31] Valiey E, Dekamin MG, Alirezvani Z (2019). Melamine-modified chitosan materials: An efficient and recyclable bifunctional organocatalyst for green synthesis of densely functionalized bioactive dihydropyrano[2,3-c]pyrazole and benzylpyrazolyl coumarin derivatives. Int. J. Biol. Macromol..

[32] Dekamin MG, Azimoshan M, Ramezani L (2013). Chitosan: A highly efficient renewable and recoverable bio-polymer catalyst for the expeditious synthesis of α-amino nitriles and imines under mild conditions. Green Chem..

[33] Sadiri SM, Sadjadia A, Dekamin MG (2014). Amperometric glucose sensor based on nickel nanoparticle/chitosan and multiwall carbon nanotube on modified graphite electrode. Am. J. Anal. Chem..

[34] de Abrantes PG (2022). The efficient Knoevenagel condensation promoted by bifunctional heterogenized catalyst based chitosan-EDTA at room temperature. Catal. Lett..

[35] Lv D, Zhang M (2017). O-carboxymethyl chitosan supported heterogeneous palladium and Ni catalysts for heck reaction. Molecules.

[36] Baran T, Sargin I, Kaya M, Menteş A (2016). Green heterogeneous Pd (II) catalyst produced from chitosan-cellulose micro beads for green synthesis of biaryls. Carbohyd. Polym..

[37] Zeng M, Yuan X, Yang Z, Qi C (2014). Novel macroporous palladium cation crosslinked chitosan membranes for heterogeneous catalysis application. Int. J. Biol. Macromol..

[38] Zeng M, Qi C, Zhang X-M (2013). Chitosan microspheres supported palladium heterogeneous catalysts modified with pearl shell powders. Int. J. Biol. Macromol..

[39] Karimi B, Enders D (2006). New N-heterocyclic carbene palladium complex/ionic liquid matrix immobilized on silica: Application as recoverable catalyst for the Heck reaction. Org. Lett..

[40] Gotthardt MA, Beilmann A, Schoch R, Engelke J, Kleist W (2013). Post-synthetic immobilization of palladium complexes on metal–organic frameworks—A new concept for the design of heterogeneous catalysts for Heck reactions. RSC Adv..

[41] Lubell, W. D., Beauregard, K. S. & Polyak, F. in *Comprehensive Chirality* (eds Erick M. Carreira & Hisashi Yamamoto) 86–104 (Elsevier, 2012).

[42] Mulzer, J. in *Comprehensive Chirality* (eds Erick M. Carreira & Hisashi Yamamoto) 122–162 (Elsevier, 2012).

[43] Bossi A, Cretich M, Righetti PG (1998). Production of D-phenylglycine from racemic (D, L)-phenylglycine via isoelectrically-trapped penicillin G acylase. Biotechnol. Bioeng..

[44] Grishin D, Zhdanov D, Pokrovskaya M, Sokolov N (2020). D-amino acids in nature, agriculture and biomedicine. All Life.

[45] Tao G-H (2006). Preparation, characterization and application of amino acid-based green ionic liquids. Green Chem..

[46] Jiang Y-Y (2008). Tetraalkylammonium amino acids as functionalized ionic liquids of low viscosity. Chem. Commun..

[47] Wang X, Akhmedov NG, Duan Y, Luebke D, Li B (2013). Immobilization of amino acid ionic liquids into nanoporous microspheres as robust sorbents for CO_2_ capture. J. Mater. Chem. A.

[48] Marshall RJ (2016). Amino acids as highly efficient modulators for single crystals of zirconium and hafnium metal–organic frameworks. J. Mater. Chem. A.

[49] Stone, R. (American Association for the Advancement of Science, 2009).

[50] Kryukov GV (2003). Characterization of mammalian selenoproteomes. Science.

[51] Wang J (2017). Microwave-assisted synthesis, structure and anti-tumor activity of selenized Artemisia sphaerocephala polysaccharide. Int. J. Biol. Macromol..

[52] Gallego-Gallegos M, Doig LE, Tse JJ, Pickering IJ, Liber K (2013). Bioavailability, toxicity and biotransformation of selenium in midge (*Chironomus dilutus*) larvae exposed via water or diet to elemental selenium particles, selenite, or selenized algae. Environ. Sci. Technol..

[53] Kaur N (2018). Ultrasound-assisted green synthesis of five-membered O-and S-heterocycles. Synth. Commun..

[54] Banerjee B (2017). Recent developments on ultrasound-assisted one-pot multicomponent synthesis of biologically relevant heterocycles. Ultrason. Sonochem..

[55] Martins MA (2004). 4-Alkoxy-1,1,1-trichloro-3-alken-2-ones: Preparation and applications in heterocyclic synthesis. Curr. Org. Synth..

[56] Druzhenko T (2018). Photochemical synthesis of 2-azabicyclo [3.2.0] heptanes: Advanced building blocks for drug discovery. Synthesis of 2,3-ethanoproline. J. Org. Chem..

[57] Candeias NR, Branco LC, Gois PM, Afonso CA, Trindade AF (2009). More sustainable approaches for the synthesis of N-based heterocycles. Chem. Rev..

[58] Dondoni A (2010). Heterocycles in organic synthesis: Thiazoles and triazoles as exemplar cases of synthetic auxiliaries. Org. Biomol. Chem..

[59] Lee H-K (2005). Use of furans in synthesis of bioactive compounds. Pure Appl. Chem..

[60] Dekamin MG, Kazemi E, Karimi Z, Mohammadalipoor M, Naimi-Jamal MR (2016). Chitosan: An efficient biomacromolecule support for synergic catalyzing of Hantzsch esters by CuSO_4_. Int. J. Biol. Macromol..

[61] Saleh TS, Eldebss TM, Albishri HM (2012). Ultrasound assisted one-pot, three-components synthesis of pyrimido [1,2-a] benzimidazoles and pyrazolo [3,4-b] pyridines: A new access via phenylsulfone synthon. Ultrason. Sonochem..

[62] Boukis AC, Reiter K, Frölich M, Hofheinz D, Meier MA (2018). Multicomponent reactions provide key molecules for secret communication. Nat. Commun..

[63] Koolivand M, Nikoorazm M, Ghorbani-Choghamarani A, Azadbakht R, Tahmasbi B (2021). Ni–citric acid coordination polymer as a practical catalyst for multicomponent reactions. Sci. Rep..

[64] Hussain-Khil N, Ghorbani-Choghamarani A, Mohammadi M (2021). A new silver coordination polymer based on 4,6-diamino-2-pyrimidinethiol: Synthesis, characterization and catalytic application in asymmetric Hantzsch synthesis of polyhydroquinolines. Sci. Rep..

[65] Gu Y (2012). Multicomponent reactions in unconventional solvents: State of the art. Green Chem..

[66] Gulati S, Singh R, Sangwan S (2021). Fruit juice mediated multicomponent reaction for the synthesis of substituted isoxazoles and their in vitro bio-evaluation. Sci. Rep..

[67] Ghafuri H, Tajik Z, Ghanbari N, Hanifehnejad P (2021). Preparation and characterization of graphitic carbon nitride-supported l-arginine as a highly efficient and recyclable catalyst for the one-pot synthesis of condensation reactions. Sci. Rep..

[68] Saneinezhad S, Mohammadi L, Zadsirjan V, Bamoharram FF, Heravi MM (2020). Silver nanoparticles-decorated Preyssler functionalized cellulose biocomposite as a novel and efficient catalyst for the synthesis of 2-amino-4H-pyrans and spirochromenes. Sci. Rep..

[69] Dekamin MG, Mehdipoor F, Yaghoubi A (2017). 1,3,5-Tris(2-hydroxyethyl)isocyanurate functionalized graphene oxide: A novel and efficient nanocatalyst for the one-pot synthesis of 3,4-dihydropyrimidin-2(1H)-ones. New J. Chem..

[70] Fattahi B, Dekamin MG (2023). Fe_3_O_4_/SiO_2_ decorated trimesic acid-melamine nanocomposite: A reusable supramolecular organocatalyst for efficient multicomponent synthesis of imidazole derivatives. Sci. Rep..

[71] Ghosh S, Saikh F, Das J, Pramanik AK (2013). Hantzsch 1,4-dihydropyridine synthesis in aqueous ethanol by visible light. Tetrahedron Lett..

[72] Li M, Zuo Z, Wen L, Wang S (2008). Microwave-assisted combinatorial synthesis of Hexa-substituted 1,4-dihydropyridines scaffolds using one-pot two-step multicomponent reaction followed by a S-alkylation. J. Comb. Chem..

[73] Ramish SM, Ghorbani-Choghamarani A, Mohammadi M (2022). Microporous hierarchically Zn–MOF as an efficient catalyst for the Hantzsch synthesis of polyhydroquinolines. Sci. Rep..

[74] Sainani J, Shah A, Arya V (1994). Synthesis of 4-Aryl-1, 4, 5, 6, 7, 8-hexahydro-5-oxo-2,7,7-trimethyl-quinoline-3-carboxylates and Amides. ChemInform.

[75] Mekheimer RA, Hameed AA, Sadek KU (2008). Solar thermochemical reactions: four-component synthesis of polyhydroquinoline derivatives induced by solar thermal energy. Green Chem..

[76] Ko S, Sastry M, Lin C, Yao C-F (2005). Molecular iodine-catalyzed one-pot synthesis of 4-substituted-1,4-dihydropyridine derivatives via Hantzsch reaction. Tetrahedron Lett..

[77] Karade NN, Budhewar VH, Shinde SV, Jadhav WN (2007). L-proline as an efficient organo-catalyst for the synthesis of polyhydroquinoline via multicomponent Hantzsch reaction. Lett. Org. Chem..

[78] Nasr-Esfahani M, Hoseini SJ, Montazerozohori M, Mehrabi R, Nasrabadi H (2014). Magnetic Fe_3_O_4_ nanoparticles: Efficient and recoverable nanocatalyst for the synthesis of polyhydroquinolines and Hantzsch 1,4-dihydropyridines under solvent-free conditions. J. Mol. Catal. A: Chem..

[79] Kassaee M, Masrouri H, Movahedi F (2010). ZnO-nanoparticle-promoted synthesis of polyhydroquinoline derivatives via multicomponent Hantzsch reaction. Monatsh. für Chemie-Chem. Mon..

[80] Breitenbucher JG, Figliozzi G (2000). Solid-phase synthesis of 4-aryl-1,4-dihydropyridines via the Hantzsch three component condensation. Tetrahedron Lett..

[81] Das B, Ravikanth B, Ramu R, Rao BV (2006). An efficient one-pot synthesis of polyhydroquinolines at room temperature using HY-zeolite. Chem. Pharm. Bull..

[82] Kumar P, Kumar A, Hussain K (2012). Iodobenzene diacetate (IBD) catalyzed an quick oxidative aromatization of Hantzsch-1,4-dihydropyridines to pyridines under ultrasonic irradiation. Ultrason. Sonochem..

[83] Pagadala R, Maddila S, Jonnalagadda SB (2014). Eco-efficient ultrasonic responsive synthesis of pyrimidines/pyridines. Ultrason. Sonochem..

[84] Chatel G, Varma RS (2019). Ultrasound and microwave irradiation: contributions of alternative physicochemical activation methods to Green Chemistry. Green Chem..

[85] Maury SK (2020). A facile and efficient multicomponent ultrasound-assisted “on water” synthesis of benzodiazepine ring. Mol. Divers..

[86] Gupta P, Paul S (2014). Solid acids: Green alternatives for acid catalysis. Catal. Today.

[87] Wang Y, Hou Q, Ju M, Li W (2019). New developments in material preparation using a combination of ionic liquids and microwave irradiation. Nanomaterials.

[88] Fattahi AH, Dekamin MG, Clark JH (2023). Optimization of green and environmentally-benign synthesis of isoamyl acetate in the presence of ball-milled seashells by response surface methodology. Sci. Rep..

[89] Dohendou MD, Mohammad G, Namaki D (2023). Pd@L-Asparagine-EDTA-Chitosan: A highly effective and reusable bio-based and biodegradable catalyst for Heck cross-coupling reaction under mild conditions. Nanoscale Adv..

[90] Valiey E, Dekamin MG (2022). Design and characterization of an urea-bridged PMO supporting Cu(II) nanoparticles as highly efficient heterogeneous catalyst for synthesis of tetrazole derivatives. Sci. Rep..

[91] Rostami N, Dekamin MG, Valiey E, FaniMoghadam H (2022). l-Asparagine–EDTA–amide silica-coated MNPs: A highly efficient and nano-ordered multifunctional core–shell organocatalyst for green synthesis of 3,4-dihydropyrimidin-2(1H)-one compounds. RSC Adv..

[92] Nikooei N, Dekamin MG, Valiey E (2020). Benzene-1,3,5-tricarboxylic acid-functionalized MCM-41 as a novel and recoverable hybrid catalyst for expeditious and efficient synthesis of 2,3-dihydroquinazolin-4(1H)-ones via one-pot three-component reaction. Res. Chem. Intermed..

[93] Yaghoubi A, Dekamin MG (2017). Green and facile synthesis of 4H-pyran scaffold catalyzed by pure nano-ordered periodic mesoporous organosilica with isocyanurate framework (PMO-ICS). ChemistrySelect.

[94] Akbarzadeh A, Dekamin MG (2017). A facile and environmentally benign polyethylene glycol 600-mediated method for the synthesis of densely functionalized 2-aminothiophene derivatives under ultrasonication. Green Chem. Lett. Rev..

[95] Matloubi Moghaddam F, Hojabri L, Dohendou M (2003). Microwave-assisted conversion of nitriles to thioamides in solvent-free condition. Synthetic Commun..

[96] Moghaddam FM, Dakamin MG (2000). Thia-Fries rearrangement of aryl sulfonates in dry media under microwave activation. Tetrahedron Lett..

[97] Moghaddam FM, Ghaffarzadeh M, Dakamin MG (2000). Microwave assisted Willgerodt-Kindler reaction of styrenes. J. Chem. Res..

[98] Clark JH, Dekamin MG, Moghaddam FM (2002). Genuinely catalytic Fries rearrangement using sulfated zirconia. Green Chem..

[99] Rao GD, Nagakalyan S, Prasad G (2017). Solvent-free synthesis of polyhydroquinoline derivatives employing mesoporous vanadium ion doped titania nanoparticles as a robust heterogeneous catalyst via the Hantzsch reaction. RSC Adv..

[100] Zhang Q, Ma X-M, Wei H-X, Zhao X, Luo J (2017). Covalently anchored tertiary amine functionalized ionic liquid on silica coated nano-Fe_3_O_4_ as a novel, efficient and magnetically recoverable catalyst for the unsymmetrical Hantzsch reaction and Knoevenagel condensation. RSC Adv..

[101] Zhaleh S, Hazeri N, Faghihi MR, Maghsoodlou MT (2016). Chitosan: A sustainable, reusable and biodegradable organocatalyst for green synthesis of 1,4-dihydropyridine derivatives under solvent-free condition. Res. Chem. Intermed..

[102] Singh SK, Singh KN (2010). Glycine-catalyzed easy and efficient one-pot synthesis of polyhydroquinolines through Hantzsch multicomponent condensation under controlled microwave. J. Heterocycl. Chem..

[103] Taghavi Fardood S, Ramazani A, Golfar Z, Joo SW (2017). Green synthesis of Ni–Cu–Zn ferrite nanoparticles using tragacanth gum and their use as an efficient catalyst for the synthesis of polyhydroquinoline derivatives. Appl. Organomet. Chem..

[104] Shiri L, Ghorbani-Choghamarani A, Kazemi M (2017). Synthesis and characterization of DETA/Cu (NO_3_)_2_ supported on magnetic nanoparticles: a highly active and recyclable catalyst for the solvent-free synthesis of polyhydroquinolines. Monatsh. für Chemie-Chem. Mont..

[105] Goel V, Bajwan A, Chauhan S, Goel S (2018). An efficient and versatile method for synthesis of 1,4-dihydropyridines at mild reaction conditions. Chem. Sci..

[106] Mirzaei H, Davoodnia A (2012). Microwave assisted sol–gel synthesis of MgO nanoparticles and their catalytic activity in the synthesis of Hantzsch 1,4-dihydropyridines. Chin. J. Catal..

[107] Debache A (2009). An efficient one-step synthesis of 1,4-dihydropyridines via a triphenylphosphine-catalyzed three-component Hantzsch reaction under mild conditions. Tetrahedron Lett..

[108] Taheri N, Heidarizadeh F, Kiasat A (2017). A new magnetically recoverable catalyst promoting the synthesis of 1,4-dihydropyridine and polyhydroquinoline derivatives via the Hantzsch condensation under solvent-free conditions. J. Magn. Magn. Mater..

[109] Niaz H (2015). Synthesis of diethyl 4-substituted-2,6-dimethyl-1,4-dihydropyridine-3,5-dicarboxylates as a new series of inhibitors against yeast α-glucosidase. Eur. J. Med. Chem..

[110] Das SK, Mondal S, Chatterjee S, Bhaumik A (2018). N-rich porous organic polymer as heterogeneous organocatalyst for the one-pot synthesis of polyhydroquinoline derivatives through the Hantzsch condensation reaction. ChemCatChem.

[111] Li BL, Zhong AG, Ying AG (2015). Novel SO_3_H-functionalized ionic liquids–catalyzed facile and efficient synthesis of polyhydroquinoline derivatives via hantzsch condensation under ultrasound irradiation. J. Heterocycl. Chem..

[112] FaniMoghadam H, Dekamin MG, Rostami N (2022). Para-Aminobenzoic acid grafted on silica-coated magnetic nanoparticles: A highly efficient and synergistic organocatalyst for on-water synthesis of 2,3-dihydroquinazolin-4(1H)-ones. Res. Chem. Intermed..

[113] Dekamin MG, Karimi Z, Farahmand M (2012). Tetraethylammonium 2-(N-hydroxycarbamoyl)benzoate: A powerful bifunctional metal-free catalyst for efficient and rapid cyanosilylation of carbonyl compounds under mild conditions. Catal. Sci. Technol..

[114] Dekamin MG, Sagheb-Asl S, Reza Naimi-Jamal M (2009). An expeditious synthesis of cyanohydrin trimethylsilyl ethers using tetraethylammonium 2-(carbamoyl)benzoate as a bifunctional organocatalyst. Tetrahedron Lett..

[115] Yoo JS, Laughlin TJ, Krob JJ, Mohan RS (2015). Bismuth (III) bromide catalyzed synthesis of polyhydroquinoline derivatives via the Hantzsch reaction. Tetrahedron Lett..

[116] Dekamin MG (2018). Alginic acid: A mild and renewable bifunctional heterogeneous biopolymeric organocatalyst for efficient and facile synthesis of polyhydroquinolines. Int. J. Biol. Macromol..

[117] Choudhury P, Ghosh P, Basu B (2020). Amine-functionalized graphene oxide nanosheets (AFGONs): an efficient bifunctional catalyst for selective formation of 1,4-dihydropyridines, acridinediones and polyhydroquinolines. Mol. Divers..

[118] Rekunge DS, Khatri CK, Chaturbhuj GU (2017). Sulfated polyborate: An efficient and reusable catalyst for one pot synthesis of Hantzsch 1,4-dihydropyridines derivatives using ammonium carbonate under solvent free conditions. Tetrahedron Lett..

[119] Safaiee M (2018). Synthesis and application of chitosan supported vanadium oxo in the synthesis of 1,4-dihydropyridines and 2,4,6-triarylpyridines via anomeric based oxidation. New J. Chem..

[120] Boskovic, G. & Baerns, M. Catalyst Deactivation. in *Basic Principles in Applied Catalysis* (ed M. Baerns) 477–503. (Springer, 2004)

