# Supplementary material for: Ultrasound-Promoted preparation and application of novel bifunctional core/shell Fe3O4@SiO2@PTS-APG as a robust catalyst in the expeditious synthesis of Hantzsch esters
Source: Sci Rep. 2023 May 17;13:8016. doi: 10.1038/s41598-023-33990-7 (PMC10192457; doi:10.1038/s41598-023-33990-7)
Supplement: Supplementary file 1 — Supplementary Information. [file 41598_2023_33990_MOESM1_ESM.pdf]

Ultrasound-Promoted preparation and application of novel bifunctional core-shell $Fe_3O_4@SiO_2@PTS$-APG as a robust catalyst in the expeditious synthesis of Hantzsch esters

Peyman Shakib, Mohammad G. Dekamin [a]*, Ehsan Valiey [a], [a] Shahriar Karami and Mohammad Dohendou [a]

[a]Pharmaceutical and Heterocyclic Compounds Research Laboratory Department of Chemistry Iran University of Science and Technology. Email: mdekamin@iust.ac.ir

Contents	Page
**Figure 1S.** Schematic preparation of the $Fe_3O_4@SiO_2@PTS$-APG nanocatalyst (**1**).	2
**Figure 2S .**Synthesis of PHQ **6** and 1,4-DHP **7** derivatives catalyzed by the $Fe_3O_4@SiO_2@PTS$-APG nanomagnetic catalyst (**1**)	2
FTIR spectra of Fe_3O_4, $Fe_3O_4@SiO_2$, $Fe_3O_4@SiO_2@CPTS$ and $Fe_3O_4@SiO_2@PTS$-APG (**1**)	3
Energy dispersive spectroscopy (EDS) pattern of $Fe_3O_4@SiO_2@PTS$-APG	4
FESEM images of the $Fe_3O_4@SiO_2@PTS$-APG nanomaterial (**1**)	4
XRD Pattern of the $Fe_3O_4@SiO_2@PTS$-APG nanocatalyst (**1**)	5
TGA curve of the $Fe_3O_4@SiO_2@PTS$-APG nanomaterial (**1**)	5
Magnetization curves of the Fe_3O_4 (red), $Fe_3O_4@SiO_2$ and $Fe_3O_4@SiO_2@PTS$-APG MNPs (**1**)	6
Reusability of the $Fe_3O_4@SiO_2@PTS$-APG MNPs **1** in the synthesis of **6b** and **7a** under optimized conditions	6
FTIR spectrum of ethyl 4-(4-(dimethylamino)phenyl)-2,7,7-trimethyl-5-oxo-1,4,5,6,7,8-hexahydroquinoline-3-carboxylate (**6a**)	7
^1H NMR spectrum of ethyl 4-(4-(dimethylamino)phenyl)-2,7,7-trimethyl-5-oxo-1,4,5,6,7,8-hexahydroquinoline-3-carboxylate (**6a**)	8
FT-IR spectrum of diethyl 4-(4-chlorophenyl)-2,6-dimethyl-1,4-dihydropyridine-3,5-dicarboxylate (**7a**)	14
^1H NMR spectrum of diethyl 4-(4-chlorophenyl)-2,6-dimethyl-1,4-dihydropyridine-3,5-dicarboxylate (**7a**)	15

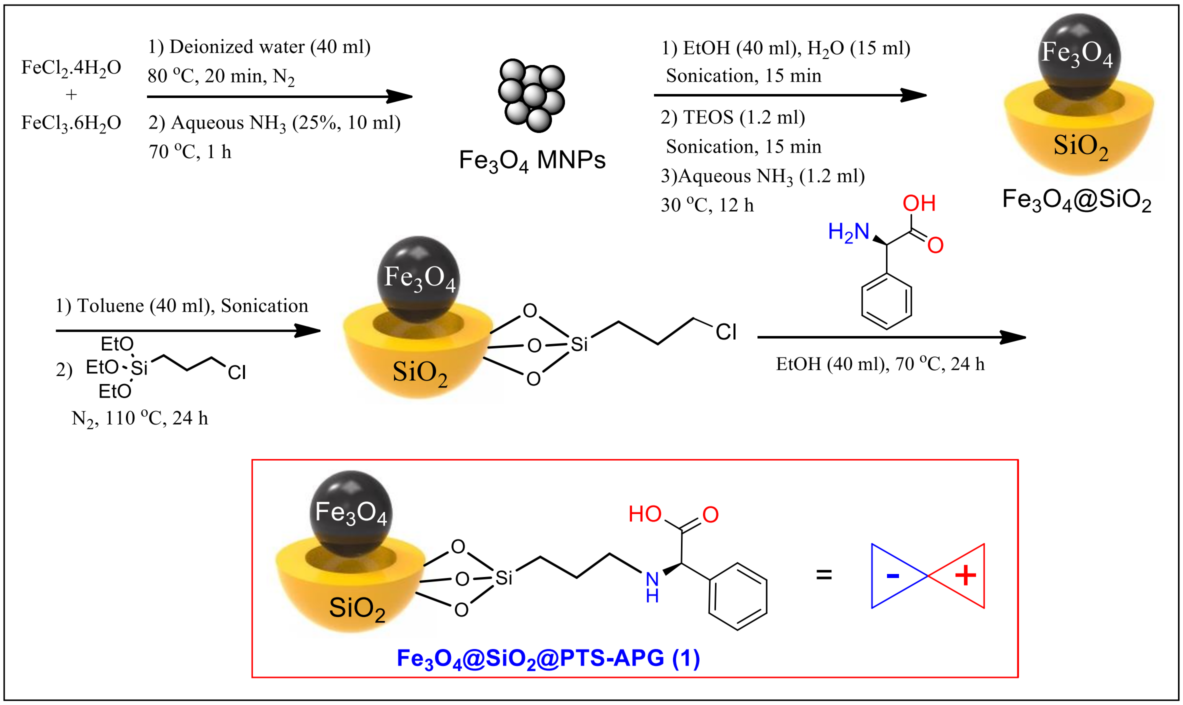

Figure 1S. Schematic preparation of the Fe_3O_4@SiO_2@PTS-APG nanocatalyst (**1**).

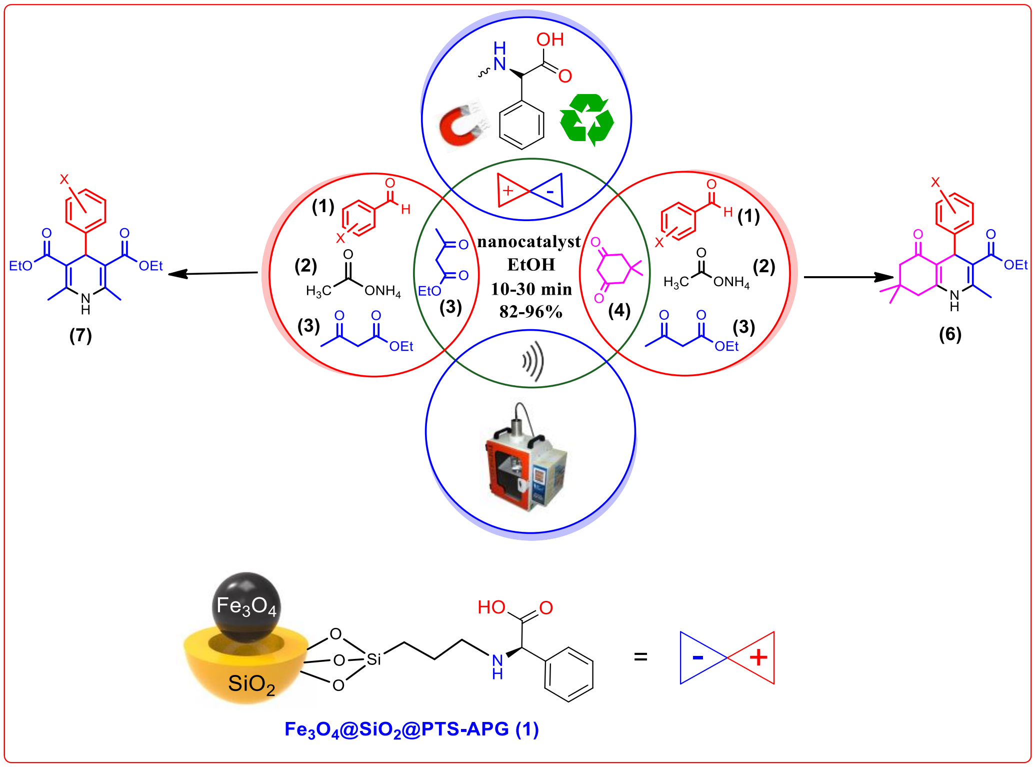

Figure 2S. Synthesis of PHQ **6** and 1,4-DHP **7** derivatives catalyzed by the Fe_3O_4@SiO_2@PTS-APG nanomagnetic catalyst (**1**).

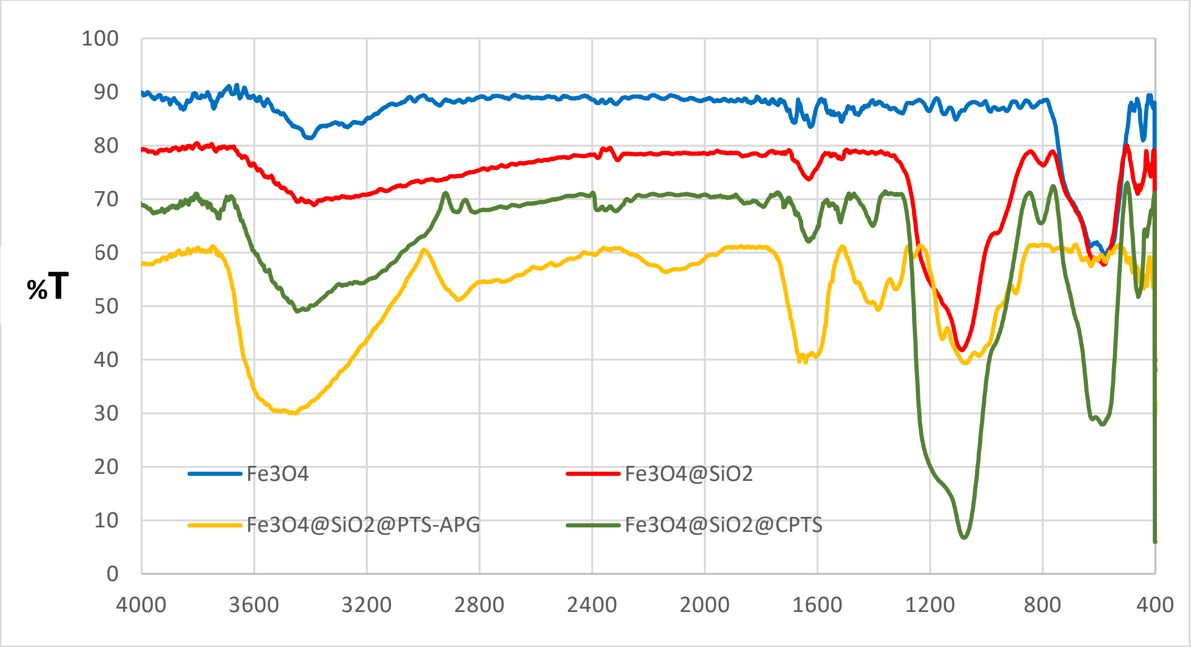

Wavenumber cm^{-1}

Figure S3. FTIR spectra of Fe_3O_4 (blue), $Fe_3O_4@SiO_2$ (red), $Fe_3O_4@SiO_2$@CPTS (green), $Fe_3O_4@SiO_2$@PTS-APG (**1**) (yellow).

Counts

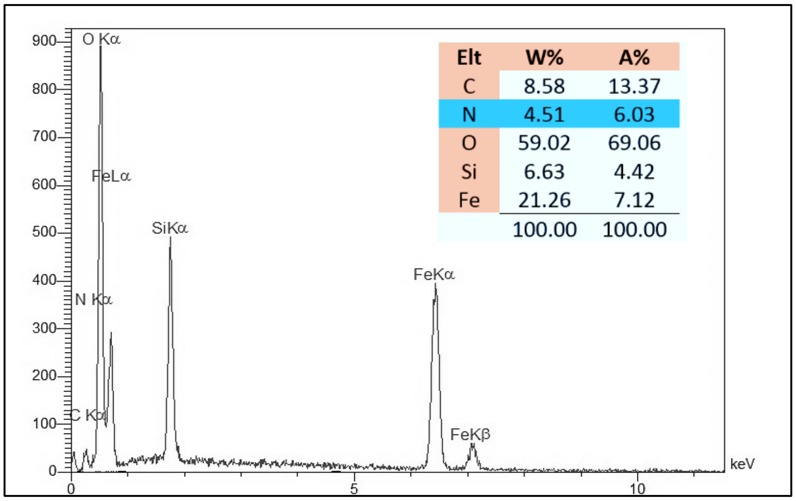

Energy (kev)

Fig. 4S. Energy dispersive spectroscopy (EDS) of the $Fe_3O_4@SiO_2$@PTS-APG catalyst (**1**).

Wavenumber (cm^{-1})

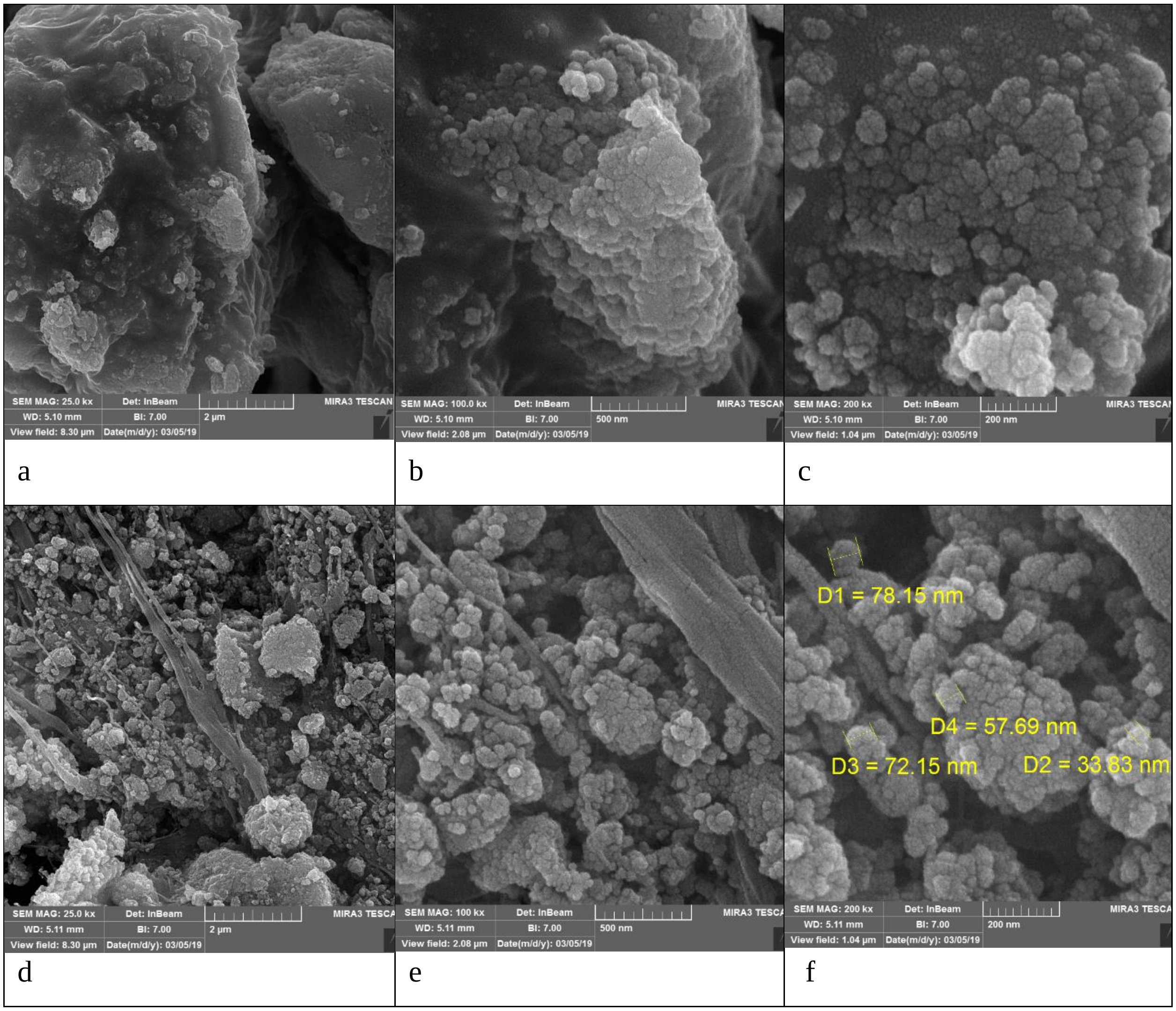

Fig. 5S. FESEM images of the Fe_3O_4@SiO_2@PTS-APG nanomaterial (**1**).

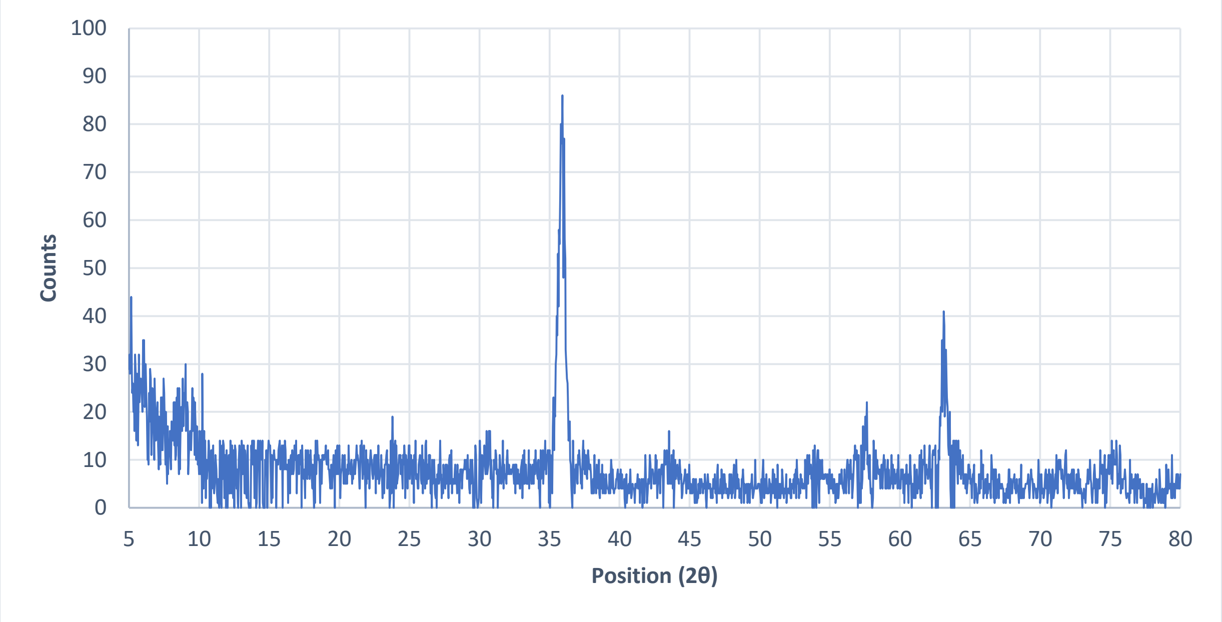

Fig. 6S. XRD Pattern of the Fe_3O_4@SiO_2@PTS-APG nanocatalyst (**1**).

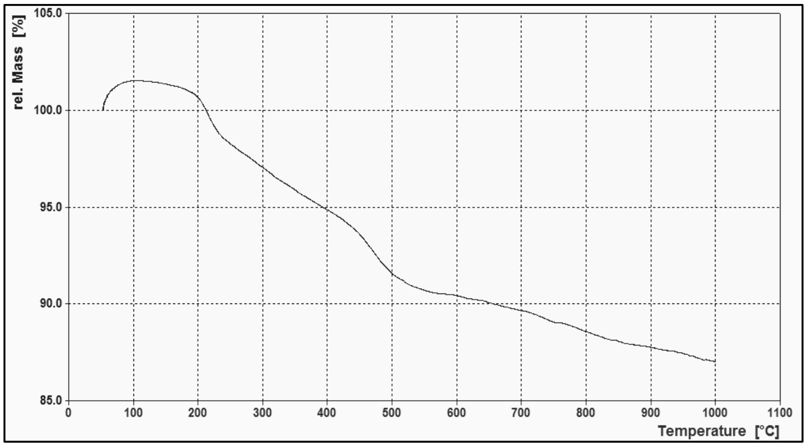

Fig. 7S. TGA curve of the Fe_3O_4@SiO_2@PTS-APG nanomaterial (**1**).

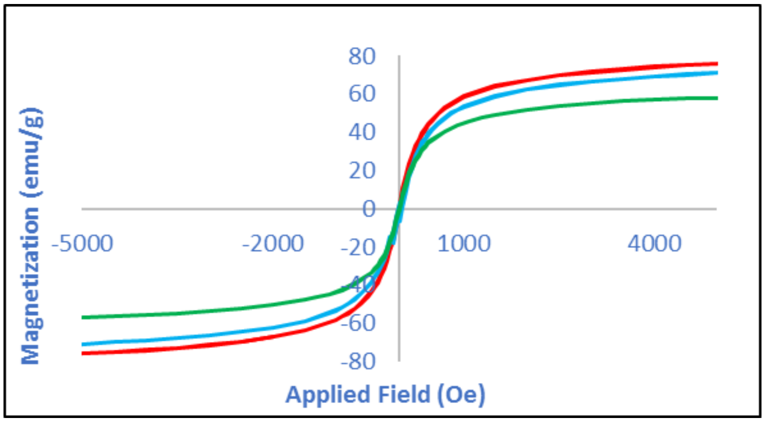

Fig. 8S Magnetization curves of the Fe_3O_4 (red), Fe_3O_4@SiO_2 (blue) and Fe_3O_4@SiO_2@PTS-APG MNPs (**1**, green).

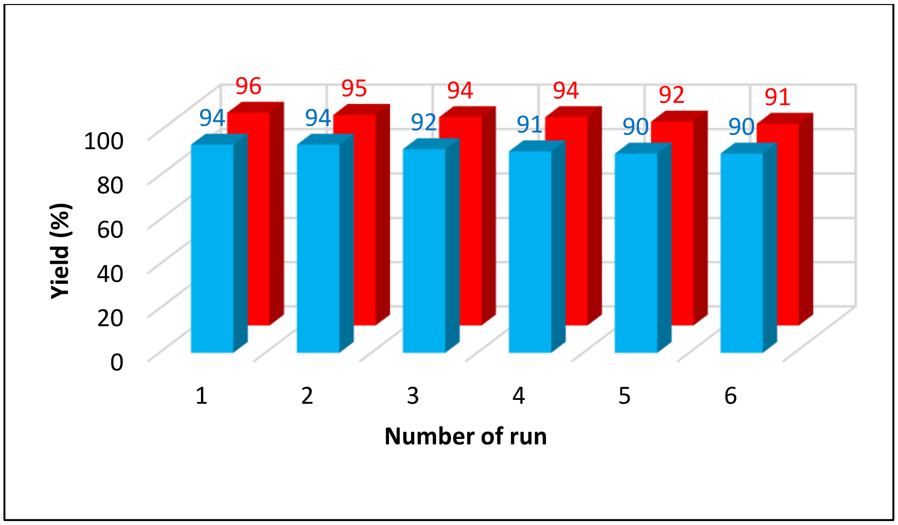

Figure 9S. Reusability of the Fe_3O_4@SiO_2@PTS-APG MNPs **1** in the synthesis of **6b** (red) and **7a** (blue) under optimized conditions.

Selected spectral data:

Ethyl 4-(4-(dimethylamino)phenyl)-2,7,7-trimethyl-5-oxo-1,4,5,6,7,8-hexahydroquinoline-3-carboxylate (**6a**)

M.p. 231-233 °C; FTIR (KBr, cm^{-1}): υ = 3282, 3205, 3076, 2956, 1704, 1606, 1490, 1380, 1278, 1213, 1107, 1072, 1029, 854, 783, 756; ^{1}H NMR ($CDCl_3$, 500 MHz): 0.94 (s, 3H), 1.06 (s, 3H), 1.17-1.20 (t, 3H, *J* = 7.3 Hz), 2.09-2.22 (m, 7H), 2.29 (s, 6H), 4.04-4.07 (q, 2H, *J* = 7.3 Hz), 5.35 (s, 1H), 6.69 (s, 1H), 7.10-7.12 (d, 2H, *J* = 7.7 Hz), 7.32-7.34 (d, 2H, *J* = 7.7 Hz).

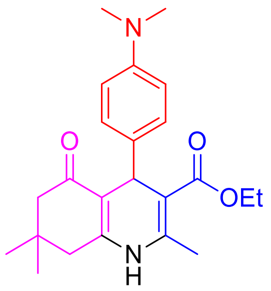

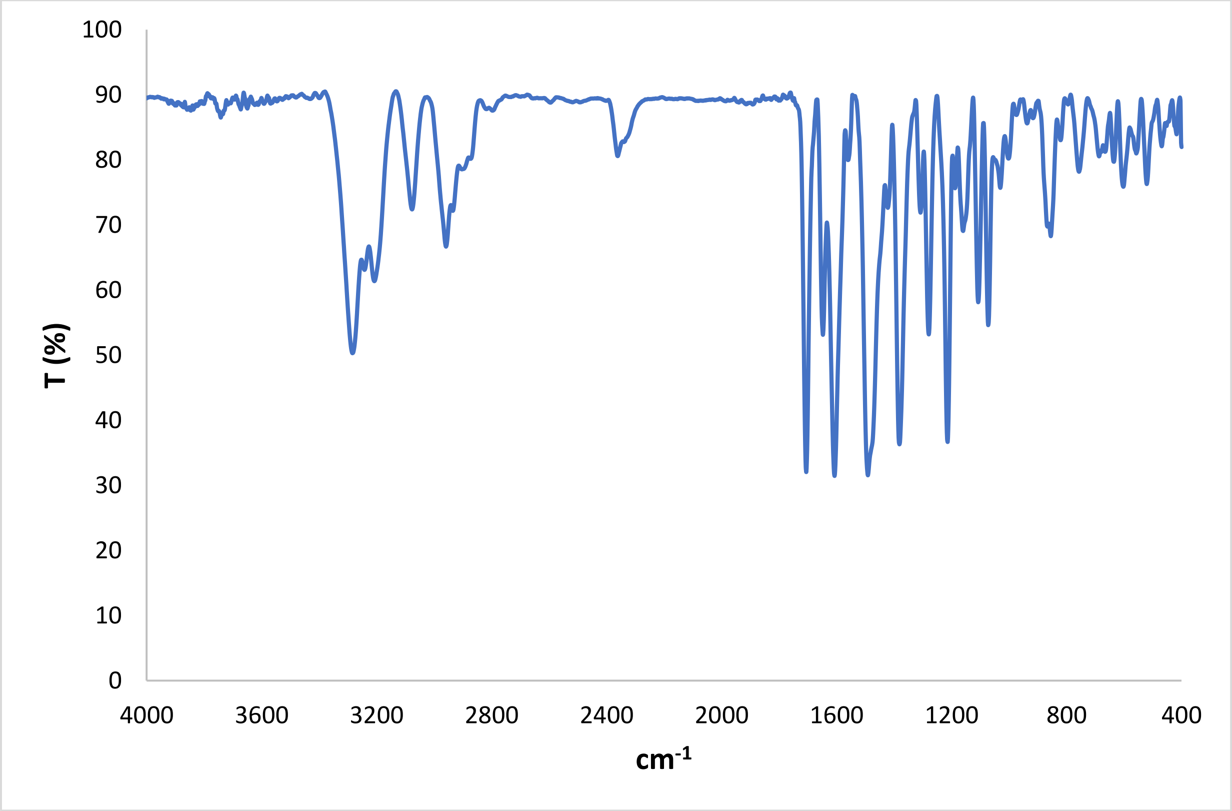

Fig. 10S. FTIR spectrum of ethyl 4-(4-(dimethylamino)phenyl)-2,7,7-trimethyl-5-oxo-1,4,5,6,7,8-hexahydroquinoline-3-carboxylate (**6a**).

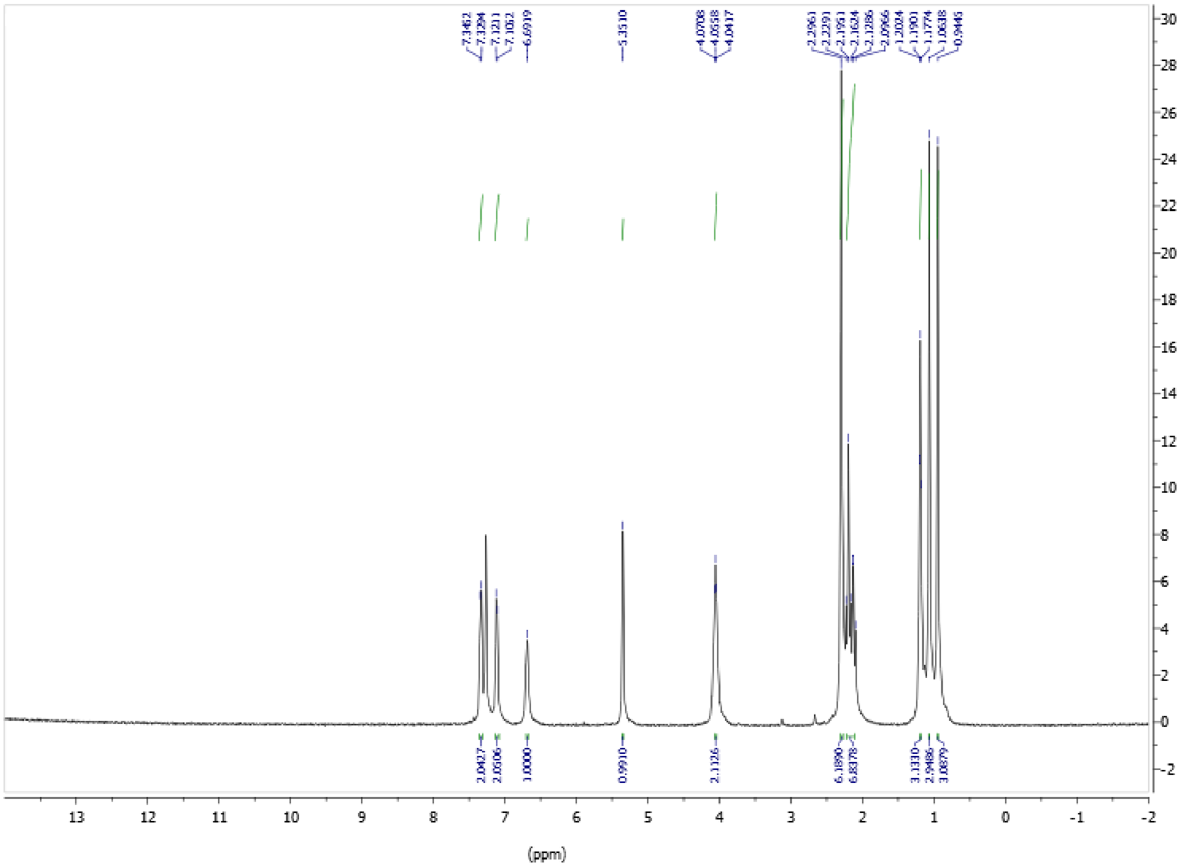

Fig. 11S. ^{1}H NMR spectrum of ethyl 4-(4-(dimethylamino)phenyl)-2,7,7-trimethyl-5-oxo-1,4,5,6,7,8-hexahydroquinoline-3-carboxylate (**6a**).

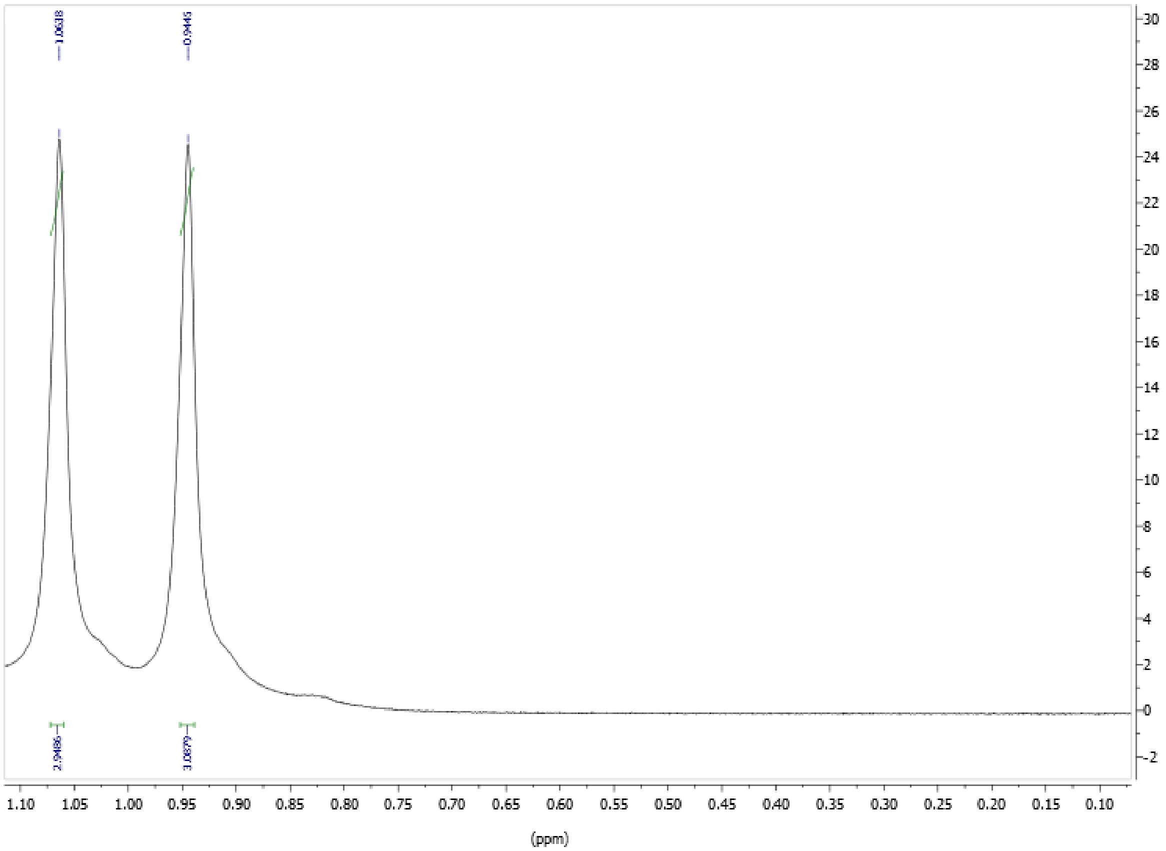

Fig. 12S. Expanded ^{1}H NMR spectrum of ethyl 4-(4-(dimethylamino)phenyl)-2,7,7-trimethyl-5-oxo-1,4,5,6,7,8-hexahydroquinoline-3-carboxylate (**6a**).

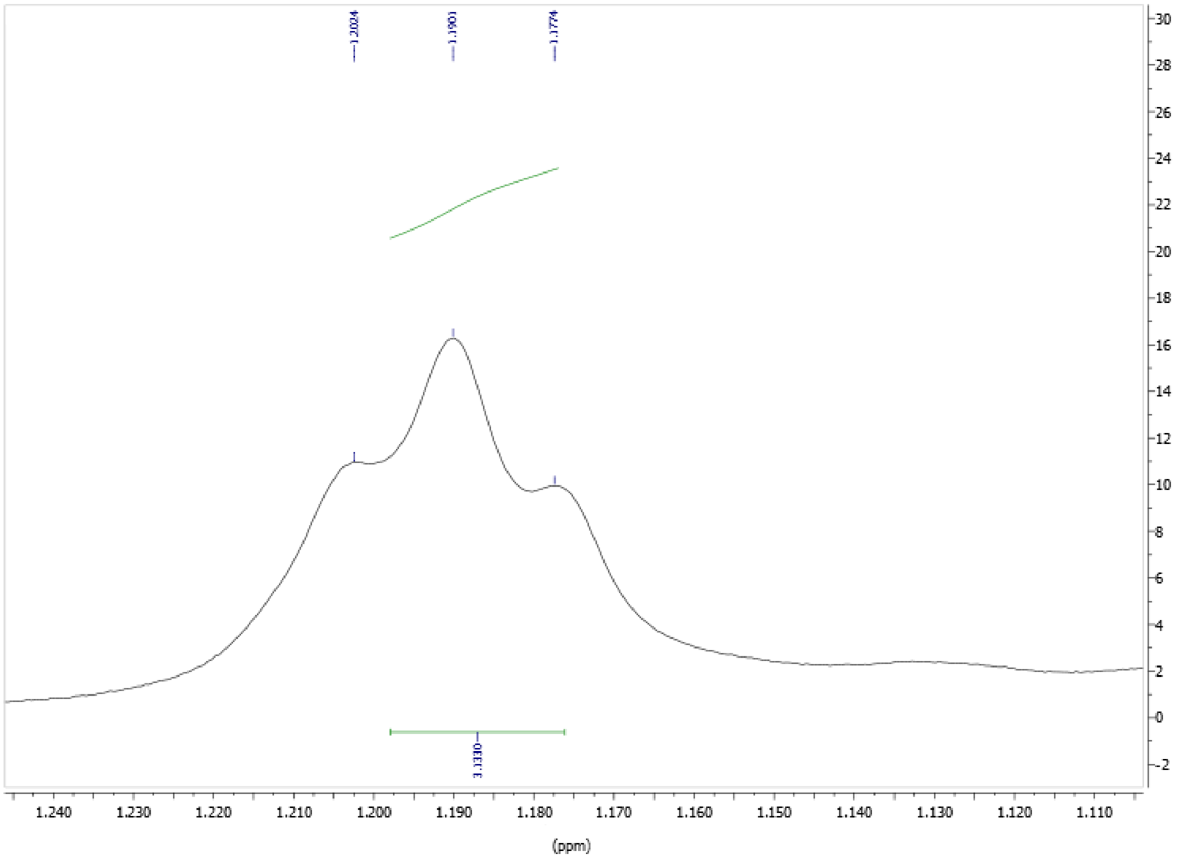

Fig. 13S. Expanded ^{1}H NMR spectrum of ethyl 4-(4-(dimethylamino)phenyl)-2,7,7-trimethyl-5-oxo-1,4,5,6,7,8-hexahydroquinoline-3-carboxylate (**6a**).

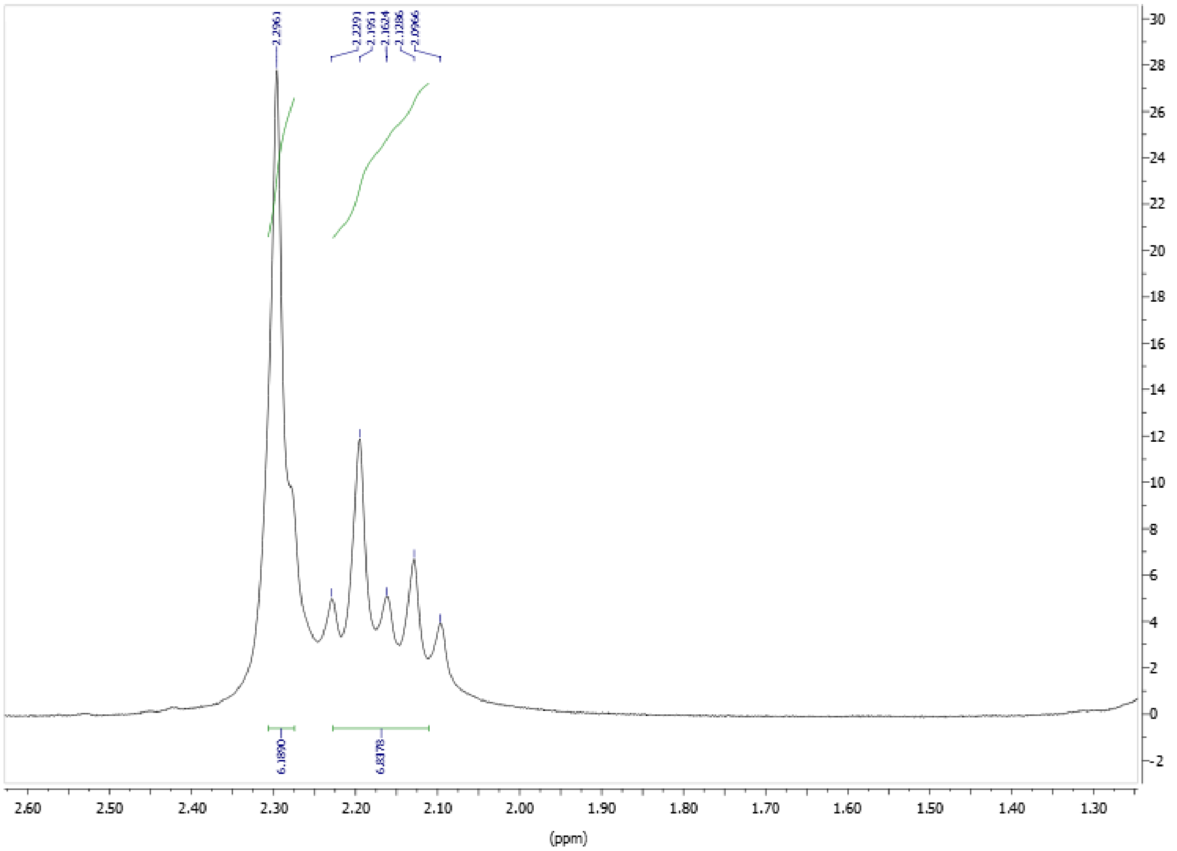

Fig. 14S. Expanded ^{1}H NMR spectrum of ethyl 4-(4-(dimethylamino)phenyl)-2,7,7-trimethyl-5-oxo-1,4,5,6,7,8-hexahydroquinoline-3-carboxylate (**6a**).

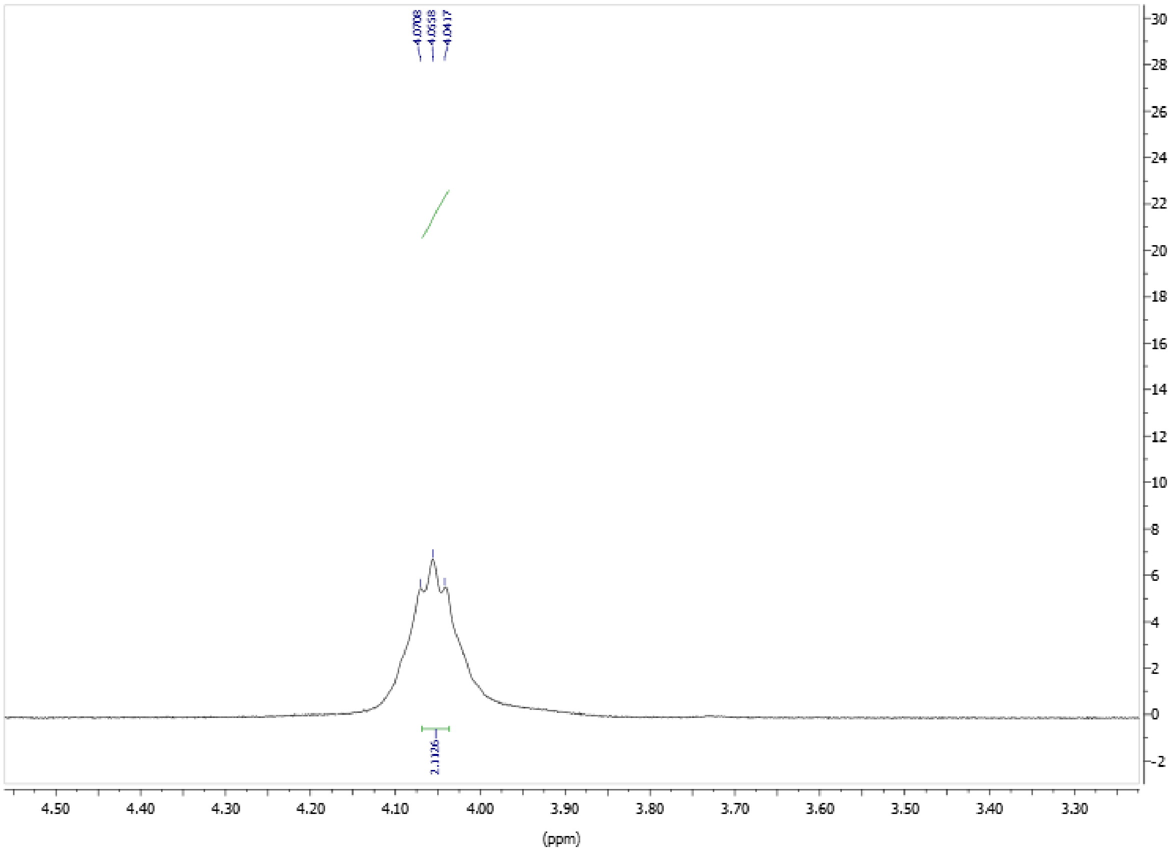

Fig. 15S. Expanded ^{1}H NMR spectrum of ethyl 4-(4-(dimethylamino)phenyl)-2,7,7-trimethyl-5-oxo-1,4,5,6,7,8-hexahydroquinoline-3-carboxylate (**6a**).

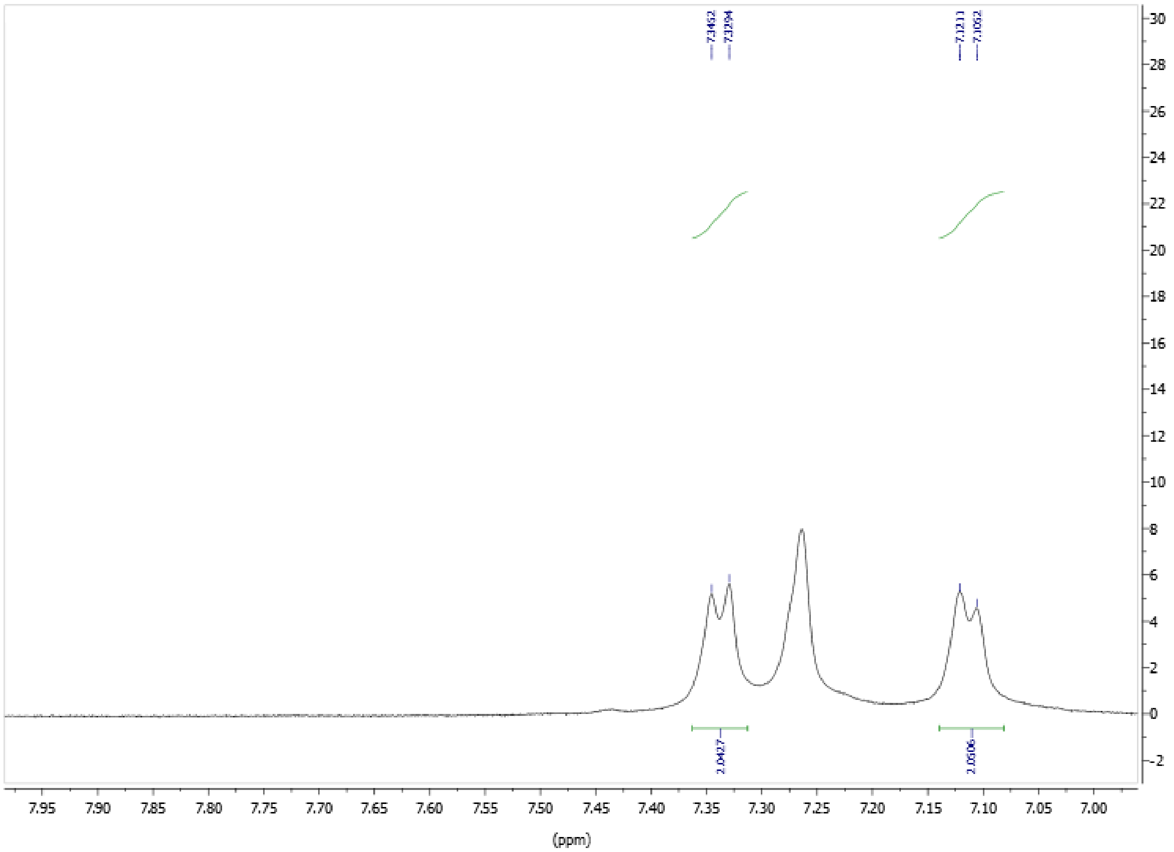

Fig. 16S. Expanded ^{1}H NMR spectrum of ethyl 4-(4-(dimethylamino)phenyl)-2,7,7-trimethyl-5-oxo-1,4,5,6,7,8-hexahydroquinoline-3-carboxylate (**6a**).

Diethyl 4-(4-chlorophenyl)-2,6-dimethyl-1,4-dihydropyridine-3,5-dicarboxylate (**7a**)

M.p. 142-144 °C; FT-IR (KBr, cm^{-1}): υ = 3355, 2985, 2958, 1697, 1649, 1487, 1371, 1292, 1213, 1091, 1051, 1016, 827, 783, 744, 669; ^{1}H NMR: ($CDCl_3$, 500 MHz): δ = 1.22-1.24 (t, 6H, *J* = 6.9 Hz), 2.34 (s, 6H), 4.09-4.11 (q, 4H, *J* = 6.9 Hz), 4.97 (s, 1H), 5.55 (s, 1H), 7.17-7.19 (d, 2H, *J* = 8.1 *Hz*), 7.21-7.22 (d, 2H, *J* = 8.1 Hz).

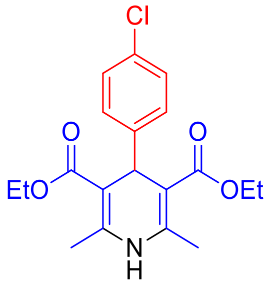

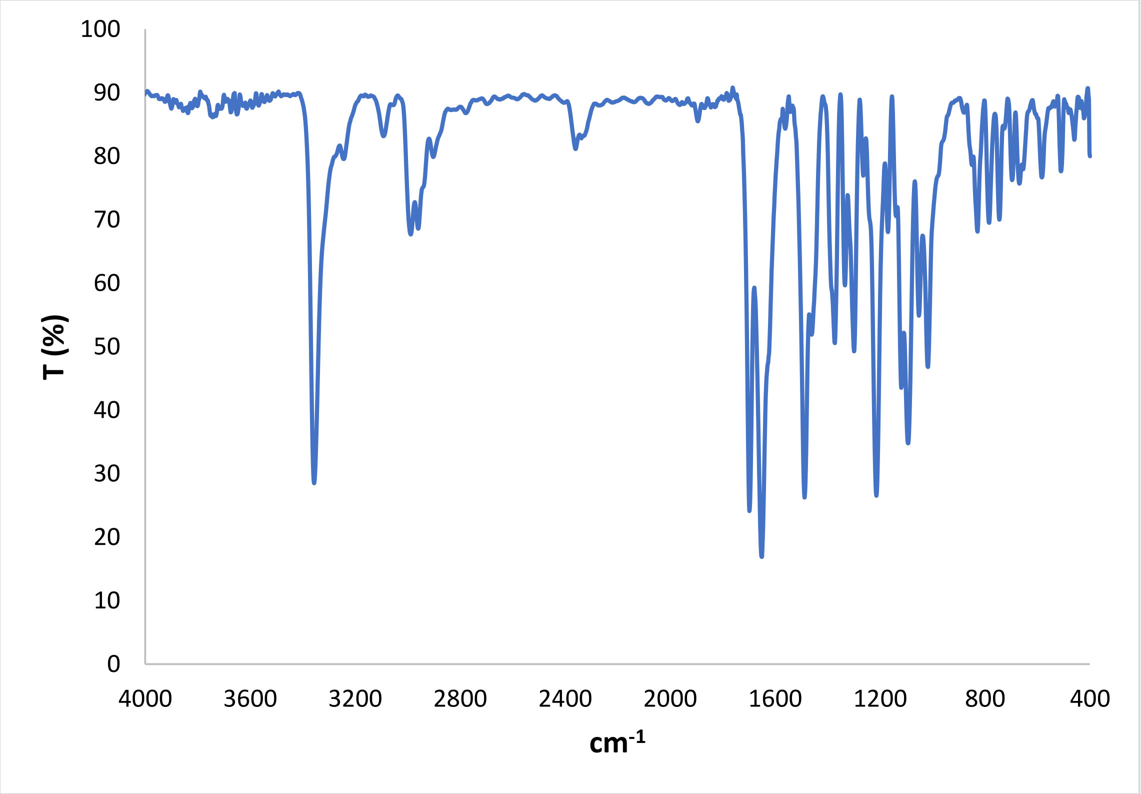

Fig. 17S. FT-IR spectrum of diethyl 4-(4-chlorophenyl)-2,6-dimethyl-1,4-dihydropyridine-3,5-dicarboxylate (**7a**).

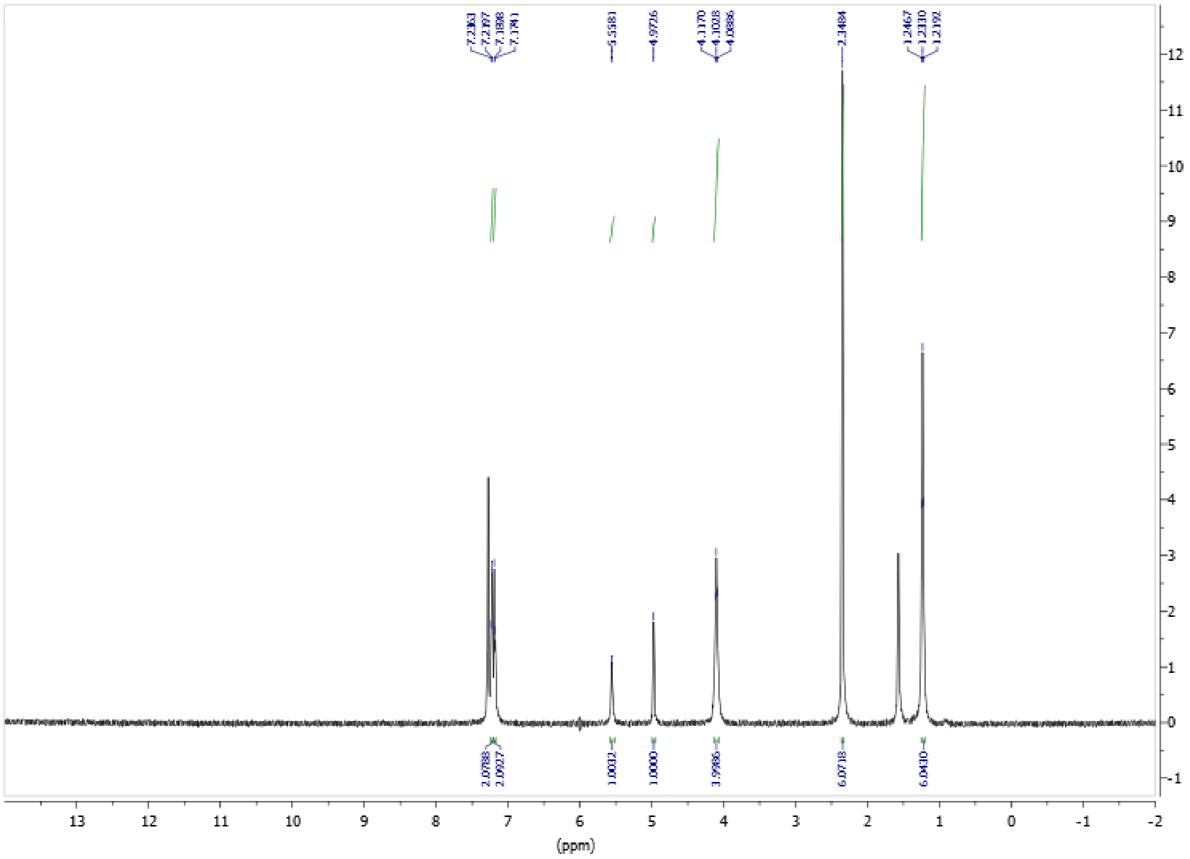

Fig. 18S. ^{1}H NMR spectrum of diethyl 4-(4-chlorophenyl)-2,6-dimethyl-1,4-dihydropyridine-3,5-dicarboxylate (**7a**).

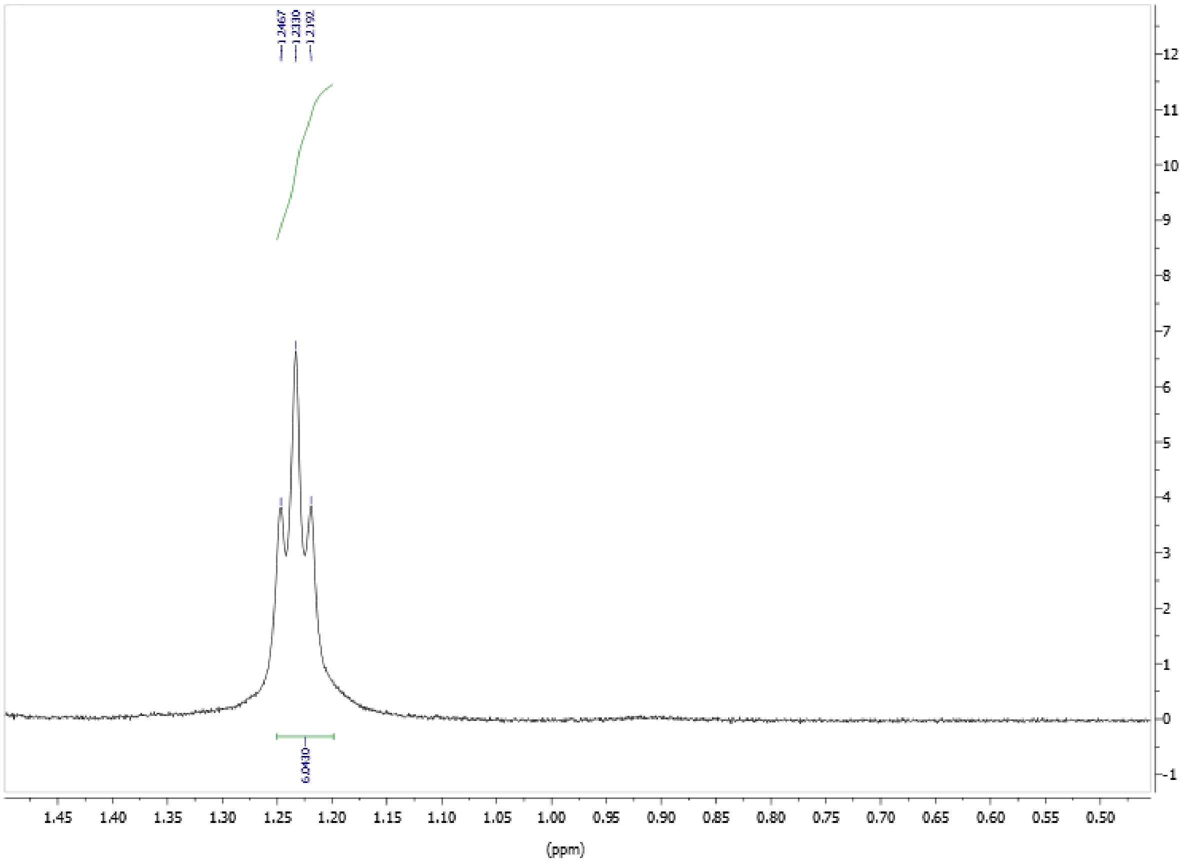

Fig. 19S. ^{1}H NMR spectrum of diethyl 4-(4-chlorophenyl)-2,6-dimethyl-1,4-dihydropyridine-3,5-dicarboxylate (**7a**).

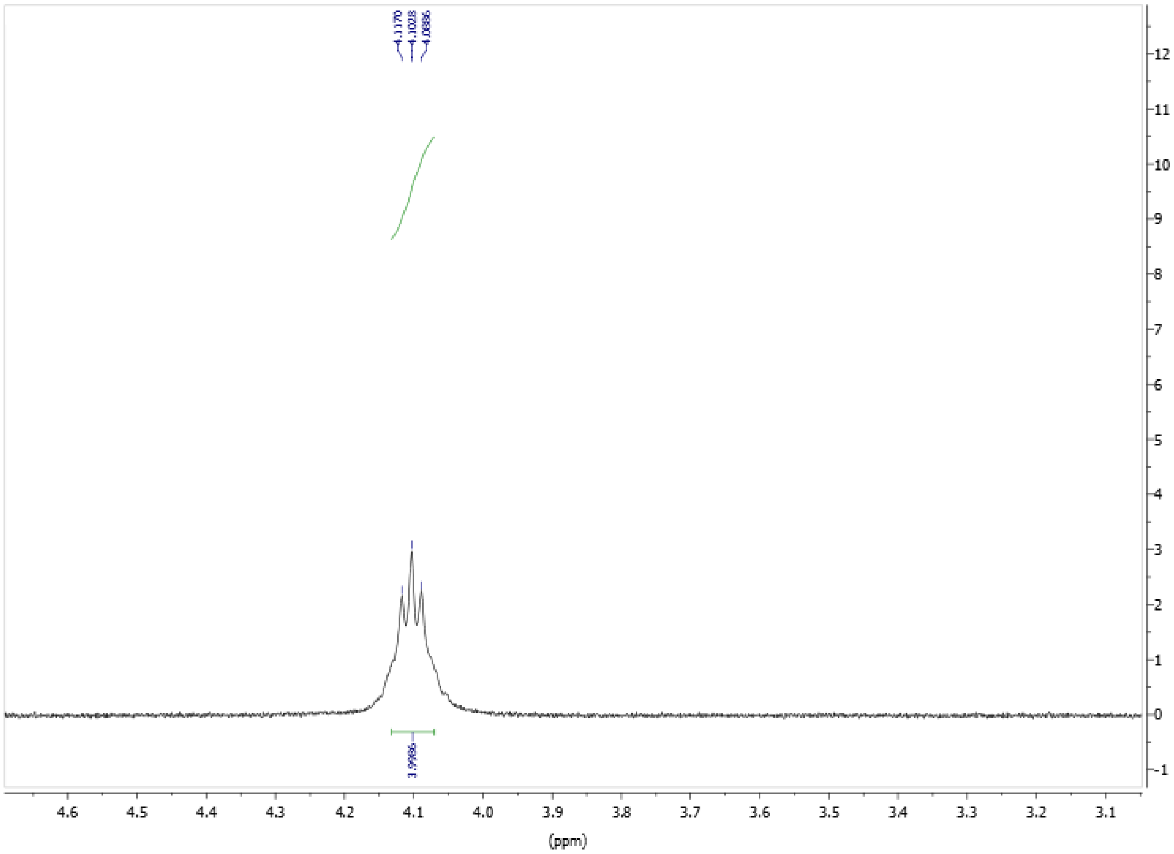

Fig. 20S. Expanded ^{1}H NMR spectrum of diethyl 4-(4-chlorophenyl)-2,6-dimethyl-1,4-dihydropyridine-3,5-dicarboxylate (**7a**).

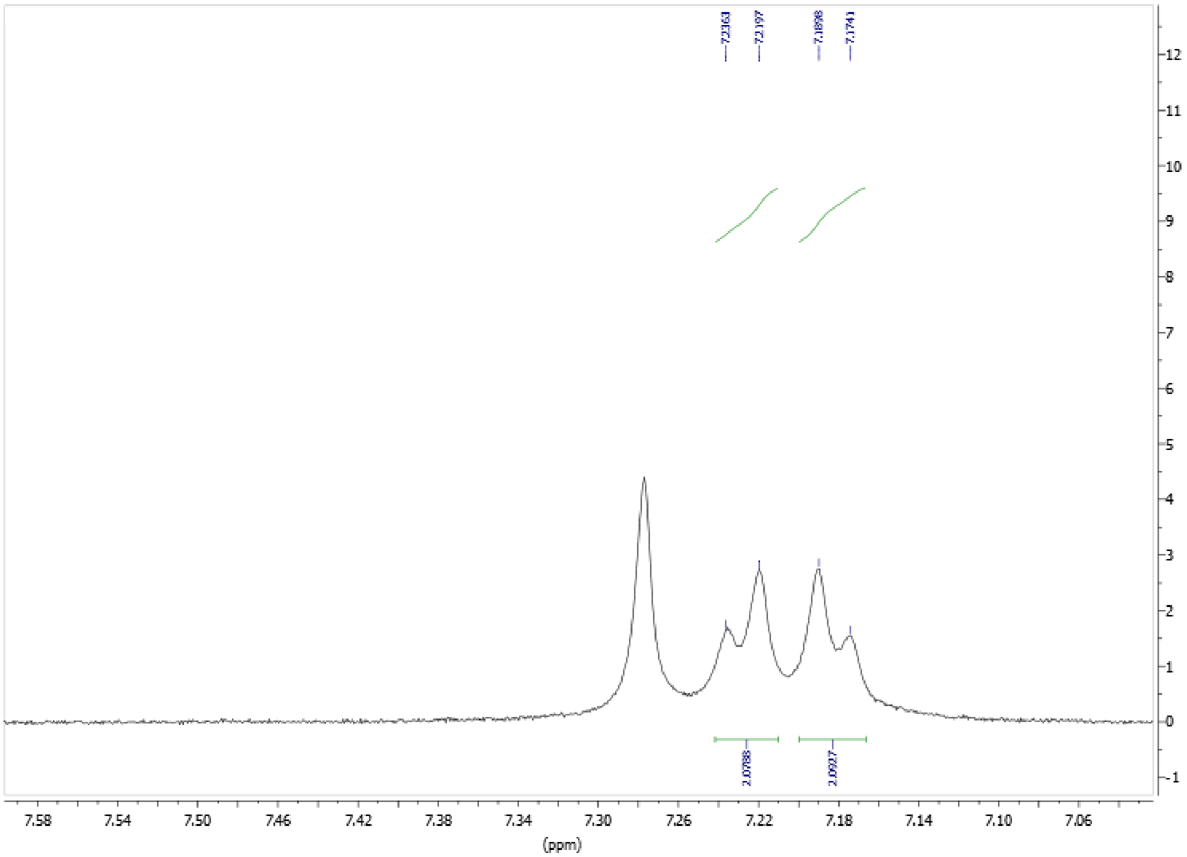

Fig. 21S Expanded ^{1}H NMR spectrum of diethyl 4-(4-chlorophenyl)-2,6-dimethyl-1,4-dihydropyridine-3,5-dicarboxylate (**7a**).
